# Preferential Binding to Elk-1 by SLE-Associated *IL10* Risk Allele Upregulates *IL10* Expression

**DOI:** 10.1371/journal.pgen.1003870

**Published:** 2013-10-10

**Authors:** Daisuke Sakurai, Jian Zhao, Yun Deng, Jennifer A. Kelly, Elizabeth E. Brown, John B. Harley, Sang-Cheol Bae, Marta E. Alarcόn-Riquelme, on behalf of the BIOLUPUS and GENLES networks, Jeffrey C. Edberg, Robert P. Kimberly, Rosalind Ramsey-Goldman, Michelle A. Petri, John D. Reveille, Luis M. Vilá, Graciela S. Alarcón, Kenneth M. Kaufman, Timothy J. Vyse, Chaim O. Jacob, Patrick M. Gaffney, Kathy Moser Sivils, Judith A. James, Diane L. Kamen, Gary S. Gilkeson, Timothy B. Niewold, Joan T. Merrill, R. Hal Scofield, Lindsey A. Criswell, Anne M. Stevens, Susan A. Boackle, Jae-Hoon Kim, Jiyoung Choi, Bernardo A. Pons-Estel, on behalf of the Argentine Collaborative Group, Barry I. Freedman, Juan-Manuel Anaya, Javier Martin, C. Yung Yu, Deh-Ming Chang, Yeong Wook Song, Carl D. Langefeld, Weiling Chen, Jennifer M. Grossman, Rita M. Cantor, Bevra H. Hahn, Betty P. Tsao

**Affiliations:** 1 Division of Rheumatology, David Geffen School of Medicine, University of California Los Angeles, Los Angeles, California, United States of America; 2 Arthritis & Clinical Immunology Program, Oklahoma Medical Research Foundation, Oklahoma City, Oklahoma, United States of America; 3 Department of Medicine, University of Alabama at Birmingham, Birmingham, Alabama, United States of America; 4 Department of Epidemiology, University of Alabama at Birmingham, Birmingham, Alabama, United States of America; 5 Division of Rheumatology and The Center for Autoimmune Genomics & Etiology, Cincinnati Children's Hospital Medical Center, Cincinnati, Ohio, United States of America; 6 US Department of Veterans Affairs Medical Center, Cincinnati, Ohio, United States of America; 7 Department of Rheumatology, Hanyang University Hospital for Rheumatic Diseases, Seoul, Korea; 8 Centro de Genómica e Investigación Oncológica (GENYO) Pfizer-Universidad de Granada-Junta de Andalucia, Granada, Spain; 9 Division of Rheumatology, Northwestern University Feinberg School of Medicine, Chicago, Illinois, United States of America; 10 Department of Medicine, Johns Hopkins University School of Medicine, Baltimore, Maryland, United States of America; 11 Department of Internal Medicine, University of Texas-Houston Health Science Center, Houston, Texas, United States of America; 12 Department of Medicine, University of Puerto Rico Medical Sciences Campus, San Juan, Puerto Rico; 13 Divisions of Genetics and Molecular Medicine and Immunology, King's College London, London, United Kingdom; 14 Department of Medicine, Keck School of Medicine, University of Southern California, Los Angeles, Los Angeles, California, United States of America; 15 Department of Medicine, University of Oklahoma Health Sciences Center, Oklahoma City, Oklahoma, United States of America; 16 Department of Medicine, Division of Rheumatology, Medical University of South Carolina, Charleston, South Carolina, United States of America; 17 Division of Rheumatology and Department of Immunology, Mayo Clinic, Rochester, Minnesota, United States of America; 18 Clinical Pharmacology Program, Oklahoma Medical Research Foundation, Oklahoma City, Oklahoma, United States of America; 19 US Department of Veterans Affairs Medical Center, Oklahoma City, Oklahoma, United States of America; 20 Rosalind Russell Medical Research Center for Arthritis, Department of Medicine, University of California San Francisco, San Francisco, California, United States of America; 21 Division of Rheumatology, Department of Pediatrics, University of Washington, and Center for Immunity and Immunotherapies, Seattle Children's Research Institute, Seattle, Washington, United States of America; 22 Division of Rheumatology, University of Colorado School of Medicine, Aurora, Colorado, United States of America; 23 Department of Medicine, Sanatorio Parque, Rosario, Argentina; 24 Department of Internal Medicine, Wake Forest School of Medicine, Winston-Salem, North Carolina, United States of America; 25 Center for Autoimmune Diseases Research, Universidad del Rosario, Bogota, Colombia; 26 Instituto de Parasitología y Biomedicina ‘López-Neyra’, CSIC, Granada, Spain; 27 Center for Molecular and Human Genetics, Research Institute at Nationwide Children's Hospital and The Ohio State University, Columbus, Ohio, United States of America; 28 National Defense Medical Center, Taipei City, Taiwan; 29 Division of Rheumatology, Seoul National University, Seoul, Korea; 30 Department of Biostatistical Sciences and Center for Public Health Genomics, Wake Forest School of Medicine, Winston-Salem, North Carolina, United States of America; 31 Department of Human Genetics, University of California Los Angeles, Los Angeles, California, United States of America; University of Oxford, United Kingdom

## Abstract

Immunoregulatory cytokine interleukin-10 (IL-10) is elevated in sera from patients with systemic lupus erythematosus (SLE) correlating with disease activity. The established association of *IL10* with SLE and other autoimmune diseases led us to fine map causal variant(s) and to explore underlying mechanisms. We assessed 19 tag SNPs, covering the *IL10* gene cluster including *IL19*, *IL20* and *IL24*, for association with SLE in 15,533 case and control subjects from four ancestries. The previously reported *IL10* variant, rs3024505 located at 1 kb downstream of *IL10*, exhibited the strongest association signal and was confirmed for association with SLE in European American (EA) (*P* = 2.7×10^−8^, OR = 1.30), but not in non-EA ancestries. SNP imputation conducted in EA dataset identified three additional SLE-associated SNPs tagged by rs3024505 (rs3122605, rs3024493 and rs3024495 located at 9.2 kb upstream, intron 3 and 4 of *IL10*, respectively), and SLE-risk alleles of these SNPs were dose-dependently associated with elevated levels of *IL10* mRNA in PBMCs and circulating IL-10 protein in SLE patients and controls. Using nuclear extracts of peripheral blood cells from SLE patients for electrophoretic mobility shift assays, we identified specific binding of transcription factor Elk-1 to oligodeoxynucleotides containing the risk (G) allele of rs3122605, suggesting rs3122605 as the most likely causal variant regulating *IL10* expression. Elk-1 is known to be activated by phosphorylation and nuclear localization to induce transcription. Of interest, phosphorylated Elk-1 (p-Elk-1) detected only in nuclear extracts of SLE PBMCs appeared to increase with disease activity. Co-expression levels of p-Elk-1 and IL-10 were elevated in SLE T, B cells and monocytes, associated with increased disease activity in SLE B cells, and were best downregulated by ERK inhibitor. Taken together, our data suggest that preferential binding of activated Elk-1 to the *IL10* rs3122605-G allele upregulates *IL10* expression and confers increased risk for SLE in European Americans.

## Author Summary

Systemic lupus erythematosus (SLE), a debilitating autoimmune disease characterized by the production of pathogenic autoantibodies, has a strong genetic basis. Variants of the *IL10* gene, which encodes cytokine interleukin-10 (IL-10) with known function of promoting B cell hyperactivity and autoantibody production, are associated with SLE and other autoimmune diseases, and serum IL-10 levels are elevated in SLE patients correlating with increased disease activity. In this study, to discover SLE-predisposing causal variant(s), we assessed variants within the genomic region containing *IL10* and its gene family member *IL19*, *IL20* and *IL24* for association with SLE in case and control subjects from diverse ancestries. We identified SLE-associated SNP rs3122605 located at 9.2 kb upstream of *IL10* as the most likely causal variant in subjects of European ancestry. The SLE-risk allele of rs3122605 was dose-dependently associated with elevated *IL10* expression at both mRNA and protein levels in peripheral blood samples from SLE patients and controls, which could be explained, at least in part, by its preferential binding to Elk-1, a transcription factor activated in B cells during active disease of SLE patients. Elk-1-mediated IL-10 overexpression could be downregulated by inhibiting activation of mitogen-activated protein kinases, suggesting a potential therapeutic target for SLE.

## Introduction

The gene cluster that includes interleukin 10 (*IL10*), *IL19*, *IL20* and *IL24* is located on chromosome 1q31-32, a genomic region that is linked with susceptibility to systemic lupus erythematosus (SLE, OMIM 152700) [1], [2]. Recent genome-wide association (GWA) and follow-up replication studies in European ancestry have identified an association between the minor allele of rs3024505, a SNP located at 1 kb downstream of *IL10*, and increased risk for SLE [3], inflammatory bowel disease (IBD) including both Crohn's disease (CD) [4] and ulcerative colitis (UC) [5], [6], and decreased risk for type 1 diabetes [7], [8]. In addition, SNPs in the second intron (rs1518111) and the promoter region (rs1800871) of *IL10* have been reported to be associated with Behçet's disease (BD) in GWAS of Turks [9] and Japanese [10]. These findings indicate *IL10* as a common susceptibility locus shared by SLE and several other autoimmune diseases.

Dysregulation of IL-10 family cytokines contributes to autoimmune disease and tissue damage (reviewed in [11]). IL-10 is an important immunoregulatory cytokine with a wide variety of functions in T cells, B cells, natural killer cells, dendritic cells and macrophages [12]. The observations of elevated serum IL-10 levels in SLE patients correlating with increased disease activity [13], [14], and promising findings of anti-IL-10 monoclonal antibody treatment in patients with SLE [15] support a pivotal role for IL-10 in the pathogenesis of SLE. Of interest, elevated IL-10 levels were also reported in first-degree relatives of SLE patients [16], [17], suggesting that levels of *IL10* expression may be determined genetically.

In this study, we fine mapped the *IL10* gene cluster for genetic association with SLE in 15,533 case and control subjects from four diverse ancestries, identified a causal variant rs3122605 at *IL10* 5′ upstream using both genetic and functional assays, and explored the underlying molecular mechanism in explaining the elevated IL-10 levels in patients with SLE associated with increased disease activity.

## Results

### Association of four *IL10* SNPs with SLE susceptibility in European Americans

**Figure 1:**
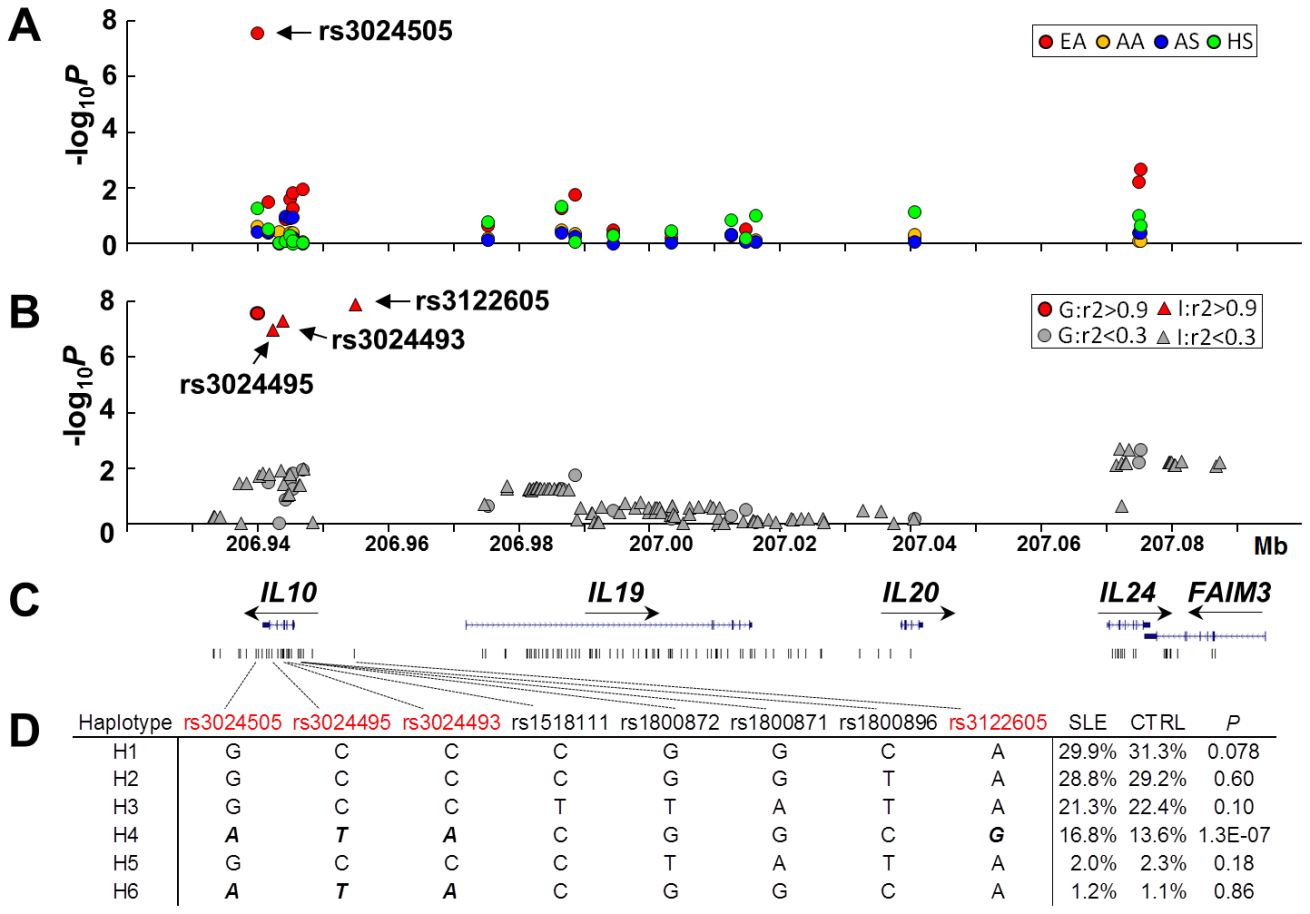
SNPs of the *IL10* gene cluster associated with SLE in European Americans. (A) Association of 19 genotyped SNPs with SLE in EA (red), AA (yellow), AS (blue) and HS (green). Allelic *P* value (−log_10_ *P*) of each SNP was plotted against its genomic position. (B) Association of 19 genotyped and an additional 109 imputed SNPs with SLE in EA. Genotyped and imputed SNPs were indicated as circles and triangles, respectively. Based on its pairwised LD strength with rs3024505, each SNP was highlighted as red (r^2^>0.9) or grey (r^2^<0.9). (C) Genomic structure of the *IL10* gene cluster and the location of each SNP. (D) Haplotypic analysis in EA. Haplotypes were constructed using four SLE-associated SNPs shown in Figure 1B (rs3024505, rs3024495, rs3024493 and rs3122605), three previously reported SLE-associated SNPs (rs1800872, rs1800871 and rs1800896 in the promoter of *IL10*) and rs1518111 (the T allele associated with Bechet's disease). Risk alleles of four SLE-associated SNPs shown in Figure 1B were bolded and italicized.

To fine map the *IL10* gene cluster, we genotyped 19 tag SNPs in 15,533 case and control subjects from four ancestries, including European American (EA, 3,820 cases vs. 3,412 controls), African American (AA, 1,670 vs. 1,904), Asian (AS, 1,252 vs. 1,249) and Amerindian/Hispanic (HS, 1,445 vs. 781). Each SNP was assessed for the association with SLE susceptibility under a logistic regression model adjusted for gender and global ancestry (Figure 1A). In the largest EA dataset, rs3024505 located at 1 kb downstream of *IL10* exhibited the strongest association with SLE (minor allele frequency of 18.2% in cases vs. 14.8% in controls, *P* = 2.7×10^−8^, OR [95%CI] = 1.30 [1.19–1.43]), which exceeded the GWAS significance level of *P*<5×10^−8^ (Table S1). To identify additional SLE-associated SNPs, we performed SNP imputation using 1000 Genomes Project data as a reference. In EA, a total of 109 well-imputed SNPs spanning 154 kb from *IL10* downstream to *FAIM3* were assessed for association with SLE. Of them, three imputed SNPs (rs3122605, rs3024493 and rs3024495 located at 9.2 kb upstream, intron 3 and 4 of *IL10*, respectively), which were in tight linkage disequilibrium (LD, r^2^>0.9) with the genotyped SNP rs3024505, were strongly associated with SLE and remained significant after Bonferroni correction (rs3122605: *P* = 1.3×10^−8^, OR = 1.34 [1.21–1.48]; rs3024493: *P* = 5.0×10^−8^, OR = 1.29 [1.18–1.42]); rs3024495: *P* = 1.0×10^−7^, OR = 1.29 [1.17–1.41]) (Figure 1B and Table S1). None of the genotyped or imputed SNPs was significantly associated with SLE in three non-European datasets including AS, AA and HS after Bonferroni correction (Table S2). These data confirmed that *IL10* is a risk locus for SLE in EA, and thus we subsequently focused on EA only to identify the causal variant(s).

*IL10* promoter SNPs rs1800872 (also named as −592T/G), rs1800871 (−819G/A) and rs1800896 (−1082T/C), which were identified to be associated with elevated IL-10 production and SLE susceptibility in some, but not all of previous studies (reviewed in [18], [19]), showed nominal association with SLE in our EA dataset (Table S1). The BD-associated SNP rs1518111 showing effect on decreased IL-10 levels [9] was not associated with SLE in EA (Table S1). We performed haplotypic analysis to investigate relationships between these four previously reported SNPs and rs3024505, rs3024495, rs3024493 and rs3122605. Only the haplotype H4 carrying risk alleles of rs3024505, rs3024495, rs3024493 and rs3122605 was strongly associated with SLE (frequency of 16.8% in cases vs. 13.6% in controls, *P* = 1.3×10^−7^) (Figure 1D).

Due to strong LD among rs3122605, rs3024493, rs3024495 and rs3024505 in EA ancestry, their associations with SLE were highly correlated and could not be distinguished from each other using the conditional haplotype-based association test (Table S1). Conditioning on rs3122605, rs3024493, rs3024495 and rs3024505, respectively, association signals (*P*<0.05) of all other SNPs within the *IL10* gene cluster were completely eliminated (Table S1), suggesting that these four SNPs within tight LD could capture all associations of the *IL10* cluster region with SLE in EA. Of note, searching +/−200 kb of *IL10* based on the 1000 Genomes Project data, we found that rs61815643 located at 10.3 kb upstream of *IL10* was also in strong LD with rs3122605 (r^2^ = 0.9) in European subjects, which suggested that this SNP might account for association signals detected within the *IL10* gene cluster. However, because the imputation quality of rs61815643 did not reach the threshold of information score >0.9, it was not included for association test in this study.

Taken together, our data provide evidence supporting *IL10* as a risk locus for SLE in EA and the underlying causal variant(s) might be or tagged by rs3122605, rs3024505, rs3024495 and rs3024493.

### Dose-dependent association between SLE-risk allele and elevated *IL10* expression levels

**Figure 2:**
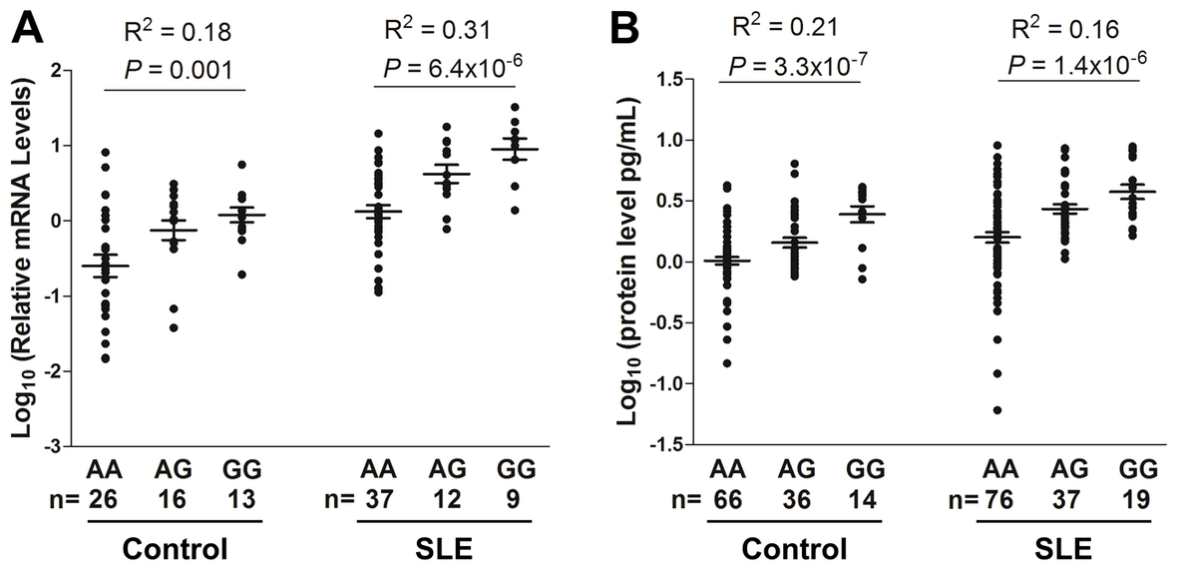
Dose-dependent association of rs3122605 risk G-allele with elevated levels of *IL10* mRNA and protein. *IL10* mRNA (A) and protein levels (B) were measured in PBMCs and plasma from EA SLE patients and healthy controls, respectively. Each symbol represents an individual and horizontal lines indicate mean ± SEM values.

To explore potential functional consequences of the SLE-associated SNPs (rs3122605, rs3024493, rs3024495 and rs3024505), we assessed their genetic effects on influencing *IL10* expression. *IL10* mRNA levels in peripheral blood mononuclear cells (PBMC) and IL-10 protein levels in plasma from EA subjects were measured by quantitative real-time PCR and ELISA, respectively. Using rs3122605 as a surrogate of the other three SNPs, we compared *IL10* expression levels among subjects carrying different genotypes of rs3122605. In control subjects, the SLE-risk (G) allele of rs3122605 was dose-dependently associated with elevated *IL10* expression at both mRNA (n = 55; *P* = 0.001, R^2^ = 0.18 in linear regression) and protein (n = 116; *P* = 3.3×10^−7^, R^2^ = 0.21) levels (Figure 2). Consistently, dose-dependent association of the risk allele with elevated *IL10* expression was also observed in patients with SLE at both mRNA (n = 58; *P* = 6.4×10^−6^, R^2^ = 0.31) and protein (n = 132; *P* = 1.4×10^−6^, R^2^ = 0.16) levels (Figure 2).

Compared to healthy controls, higher *IL10* expression was observed in patients with SLE carrying the same genotype at both mRNA (genotype AA: *P* = 1.3×10^−4^, AG: *P* = 4.3×10^−4^, GG: *P* = 3.9×10^−5^, cases vs. controls in *t* test) and protein (AA: *P* = 4.6×10^−4^, AG: *P* = 4.9×10^−6^, GG: *P* = 0.047) levels (Figure 2), probably due to the activated immune status of SLE patients.

### The risk allele of rs3122605 creates a novel binding site to transcription factor Elk-1 at 5′upstream of *IL10*

We hypothesized the presence of transcription factors activated in SLE patients upregulating *IL10* expression and prepared nuclear extracts of peripheral blood lymphocytes from active SLE patients (defined as SLEDAI score≥4) [20], [21] to perform electrophoretic mobility shift assays (EMSA) for testing allelic differences in transcription factor binding conferred by the SLE-associated SNPs rs3122605, rs3024505, rs3024495 and rs3024493. Because we could not exclude the possibility that rs61815643 is a SLE-risk SNP affecting *IL10* expression, it was also tested by EMSA.

**Figure 3:**
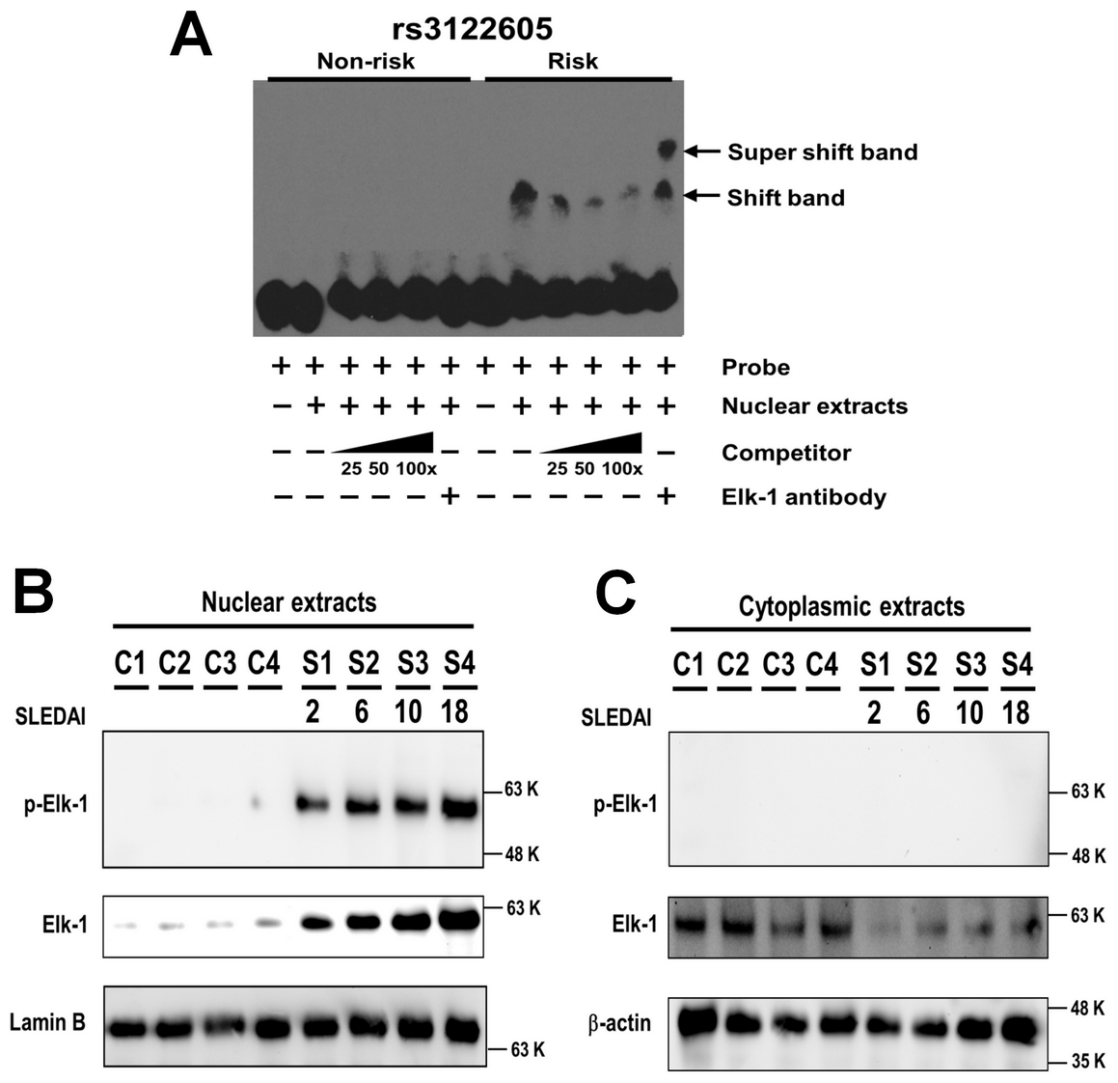
Preferential binding of rs3122605-risk allele to Elk-1, which is a transcription factor activated in peripheral lymphocytes of SLE patients. (A) Specific binding of transcription factor Elk-1 to the risk allele of rs3122605 in EMSA. Mobility-shift bands were produced by the oligodeoxynucleotide probes containing the risk allele of rs3122605 incubated with nuclear extracts of peripheral blood lymphocytes from active SLE patients, and the addition of anti-Elk-1 antibody generated a super shift band. The data are representative of two independent experiments. (B, C) Nuclear retention of activated p-Elk-1 in PBMCs from SLE patients. The presence of Elk-1 and p-Elk-1 in nuclear (B) and cytoplasmic (C) extracts of PBMCs from SLE patients and healthy controls was measured using Western blot. Lamin B (B) and β-actin (C) were used as loading controls. The data are representative of two independent experiments.

Upon incubation with nuclear extracts, specific mobility-shift bands were only detected using the oligodeoxynucleotide probe containing the risk, but not the non-risk allele of rs3122605 (Figure 3A). Of interest, no specific binding of nuclear proteins was observed with the oligodeoxynucleotide probes containing either the risk or the non-risk allele of rs3024505, rs3024493, rs3024495 and rs61815643 (Figure S1). *In silico* analysis using the program TFSEARCH indicated that the risk allele of rs3122605 might create a novel binding site of the transcription factor Elk-1 (ETS-like transcription factor 1). We validated this prediction by showing that the addition of polyclonal rabbit IgG anti-ElK-1 antibody produced a super shift band only to the probe containing the risk but not the non-risk allele of rs3122605 in EMSA (Figure 3A).

Taken together, these data showed that rs3122605, the risk allele of which binds to Elk-1 in peripheral blood lymphocytes from SLE patients with active disease, is more likely to be the causal variant upregulating *IL10* expression than the other four candidate SNPs.

### Aberrant activation of Elk-1 in the nuclei of PBMCs from patients with SLE

Elk-1 is activated through phosphorylation and the phosphorylated Elk-1 (p-Elk-1) translocates into the nucleus to induce gene transcription [22]. To investigate the role of Elk-1 in SLE, we compared the distribution and activation of Elk-1 in PBMCs between SLE cases (n = 4) and healthy controls (n = 4) using Western blot. The amount of total Elk-1 was higher in nuclear (Figure 3B) but lower in cytoplasmic extracts (Figure 3C) of cases than controls. Of interest, p-Elk-1 was detected only in nuclear extracts of cases but not in controls (Figure 3B) and not in cytoplasmic extracts of either cases or controls (Figure 3C). These data suggest that Elk-1 is aberrantly activated and accumulates in nuclei of SLE PBMCs. Furthermore, the amount of total Elk-1 and p-Elk-1 appeared to be increased in SLE patients with higher SLEDAI scores (Figure 3B).

### Co-expression of IL-10 and p-Elk-1 increases with SLE disease activity in B cells and could be best down-regulated by ERK inhibitor

**Figure 4:**
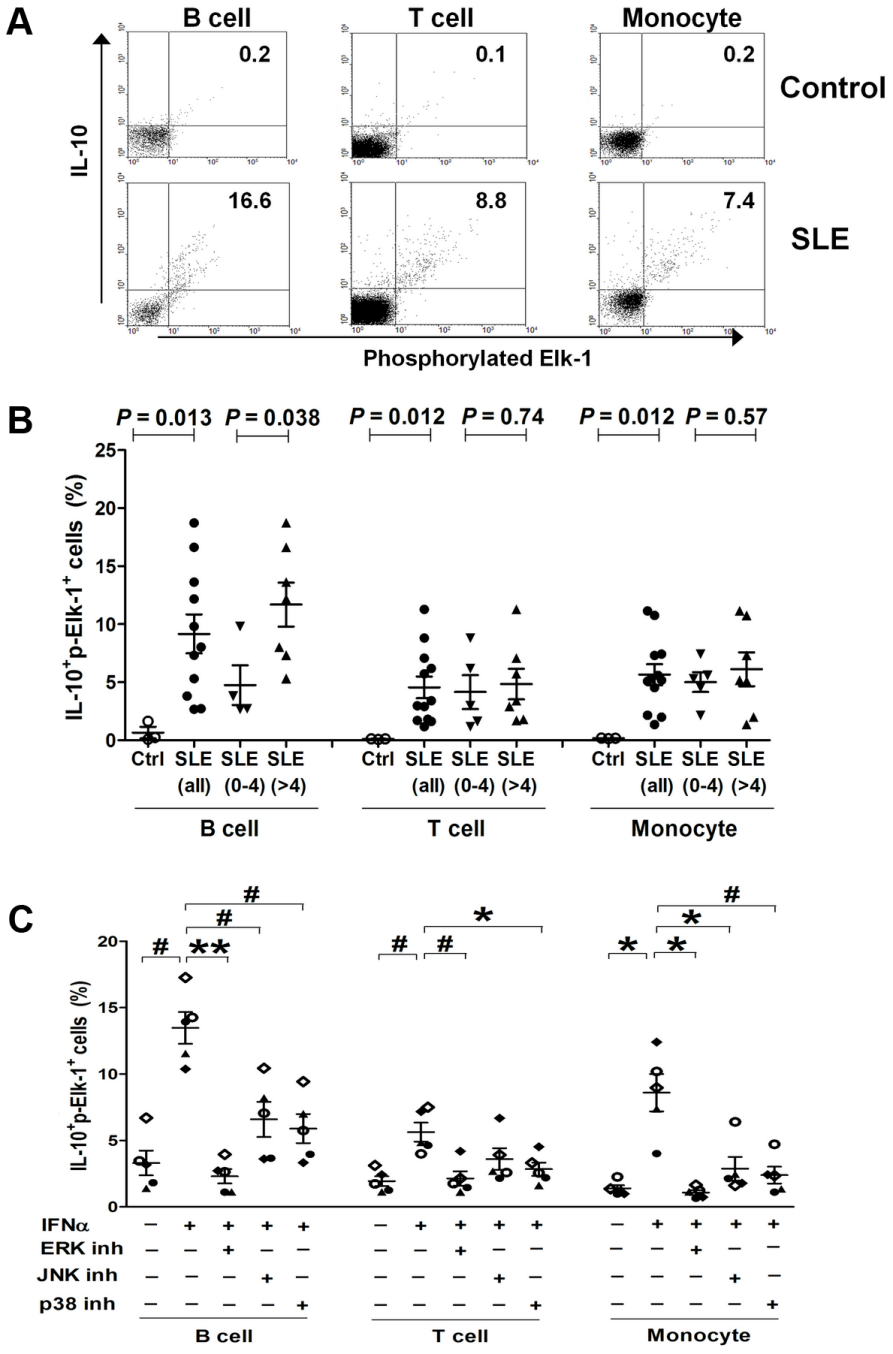
Co-expression of p-Elk-1 and IL-10 in PBMCs. (A) Quantification of co-expression of p-ELK-1 and IL-10 in B cells (CD19^+^), T cells (CD3^+^) and monocytes (CD14^+^), respectively, by flow cytometry. Numbers in upper quadrants indicate the percentages of double positive (IL-10^+^p-Elk-1^+^) cells. (B) Increased proportions of IL-10^+^p-Elk-1^+^ cells in B, T cells and monocytes from SLE patients compared to controls, and increased IL-10^+^p-Elk-1^+^ cells in B cells from active compared to inactive SLE patients. Each symbol represents an individual and horizontal lines indicate mean ± SEM values. (C) Decreased proportions of IL-10^+^p-Elk-1^+^ cells with inhibition of Elk-1 activation. Normal PBMCs were incubated with IFNα in the presence or absence of MAPK inhibitor ERK (PD 98059), JNK (SP 600125) or p38 (SB 203580), and IL-10^+^p-Elk-1^+^ cells were quantified in B, T cells and monocytes, respectively. Each symbol represents an individual and horizontal lines indicate mean ± SEM values. P*≤0.01, *P^#^*<0.005, P**<0.0001 (Student's *t* test) for the comparison of indicated groups.

Using flow cytometry, we quantified the co-expression of IL-10 and p-Elk-1 in specific cell subsets of PBMCs including CD3^+^ T cells, CD19^+^ B cells and CD14^+^ monocytes from healthy controls and SLE patients and showed representative data plotted in two-dimensional dot plots in Figure 4A. Compared to healthy controls (n = 3), percentages of IL-10^+^p-Elk-1^+^ cells were significantly increased in B (n = 11), T cells (n = 12) and monocytes (n = 12) from SLE patients (Student's *t*-test: *P* = 0.013, 0.012 and 0.012, respectively, Figure 4B). Active SLE patients had significantly elevated IL-10^+^p-Elk-1^+^ double positive B cells compared to non-active SLE patients (n = 7 vs. 4, *P* = 0.038). Similar trends for association with SLE disease activity were detected in IL-10^+^p-Elk-1^+^ T cells and monocytes, but the difference was not statistically significant (*P* = 0.74 and 0.57, respectively).

Elk-1 is known to be activated by mitogen-activated protein kinases (MAPK), including ERK (extracellular-signal-regulated kinases), JNK (c-Jun N-terminal kinases) and p38. We wondered which MAPK inhibitor could best down-regulate the elevated co-expression of IL-10 and p-Elk-1 in PBMCs. Because of the low expression of IL-10 in freshly isolated control PBMCs (Figure S2), we stimulated control PBMCs with IFN-α, a pivotal cytokine upregulated in most SLE patients [23], to mimic SLE PBMCs and incubated them with the MAPK inhibitors specific to ERK (PD 98059), JNK (SP 600125) or p38 (SB 203580), respectively. IFN-α-stimulation could significantly increase the percentage of IL-10^+^p-Elk-1^+^ cells in B, T cells and monocytes (n = 5, *P* = 0.0002, 0.0018 and 0.0079, respectively, Figure 4C), and such increase could be best down-regulated by the addition of the ERK inhibitor (*P* = 2.8×10^−5^, 0.0046 and 0.0079 in B, T cells and monocytes, respectively, Figure 4C). To a lesser extent, the p38 inhibitor also significantly suppressed IFN-α-induced double positive cells in all three cell subsets (*P* = 0.0016, 0.01 and 0.0039 in B, T cells and monocytes, respectively). Under our experimental conditions, the JNK inhibitor significantly inhibited the percentage of IL-10^+^p-Elk-1^+^ cells in B cells and monocytes (*P* = 0.0048 and 0.009, respectively), but not in T cells (*P* = 0.10).

## Discussion

Our data provide strong evidence for a dose-dependent association between SLE-predisposing *IL10* genotypes and corresponding mRNA and protein levels of IL-10, and identify one underlying molecular mechanism to explain previous findings of elevated IL-10 serum levels in SLE patients that positively correlated with increased disease activity. In addition to confirming the previously reported association with SLE at the *IL10* 3′ downstream SNP (rs3024505) in European Americans, we identified a SLE-associated risk haplotype, defined by the minor alleles of four SNPs in tight LD, rs3024505, rs3024495, rs3024493 and rs3122605, which could best explain the association with SLE and capture underlying causal variant(s) within the *IL10* gene cluster in EA ancestry. The minor allele of rs3122605, which tags the *IL10* SLE-risk haplotype, exhibited a dose-dependent association with elevated *IL10* expression at both mRNA levels in PBMCs and protein levels in plasma samples from SLE patients and healthy controls, suggesting that these four SLE-associated SNPs may act by influencing *IL10* regulation. Further functional studies showed that only rs3122605 was experimentally validated to confer preferential allele-binding to the transcription factor Elk-1 present in SLE PBMCs, hence the most likely functional variant present on the *IL10* risk haplotype. Compared to normal PBMCs, nuclear localization of activated p-Elk-1 was observed only in SLE PBMCs. Co-expression of p-Elk-1 and IL-10 was significantly increased in all SLE PBMC subsets compared to normal PBMC subsets. Of interest, SLE patients with active disease had higher double positive (p-Elk-1^+^IL-10^+^) B cells than those with inactive disease. These data suggested that nuclear accumulation of activated Elk-1 in SLE peripheral lymphocytes contributes to overproduction of IL-10 in SLE patients associated with disease activity.

An abnormally high production of IL-10 in patients with SLE has been consistently demonstrated in many studies (reviewed in [18]), but the underlying molecular mechanism remains less well-characterized. The observation that healthy relatives of SLE patients also exhibit increased levels of IL-10 [16], [24], [25] suggests a possibility of genetically regulated IL-10 production. A number of genetic polymorphisms in the *IL10* promoter region have been reported [26]–[30], in particular, three SNPs shown in Figure 1D located at −1082 (rs1800896, C/T), −819 (rs1800871, G/A) and −592 (rs1800872, G/T) have been inconsistently associated with IL-10 production levels and risk of SLE (reviewed in [18], [19]). Identification of SNPs in the −1.3 to −4 kb region of the *IL10* promoter associated with both IL-10 production phenotypes and SLE susceptibility [31] suggested that further evaluating the contribution of SNPs in the more distal promoter of *IL10* might be warranted. Consistently, the SNP (rs3122605) we identified that tags the SLE-risk haplotype in EA ancestry and confers genetic effect on *IL10* expression is located at 9.2 kb upstream of *IL10*. According to the ENCODE Project, rs3122605, rs3024505, rs3024493 and rs61815643 were located within DNasel hypersensitive and transcription factor binding sites in at least one cell type (as shown in UCSC genome browser), suggesting that each of them may affect gene expression through interaction with regulatory elements. Given that gene regulation by genetic variants often occurs within the specific cell types most relevant to the disease phenotype [32], we used nuclear extracts from PBMCs of active SLE patients to perform EMSA assays and found only the SLE-risk allele of rs3122605 preferentially binds to the transcription factor Elk-1. Therefore, our data support rs3122605 as the most likely causal variant on the SLE-associated haplotype and implicate a potential importance for Elk-1-mediated upregulation of *IL10* expression in SLE patients, particularly in those patients carrying the risk allele of rs3122605.

Elk-1 is a member of the Ets oncogene family of transcription factors characterized by a conserved DNA-binding domain and a C-terminal activation domain containing multiple phosphorylation sites targeted by three major MAP kinase pathways [33]. Different phosphorylation patterns of Elk-1 mediated through activation of MAPK signaling cascades by distinct external stimuli are important for Elk-1 to execute its physiologic functions [34]–[37]. We used an antibody to measure phosphorylation of Elk-1 at S383, and detected the presence of Elk-1 pS383 in the nuclei of SLE but not normal PBMCs. Quantification of IL-10 and p-Elk-1 co-expression confirmed a higher proportion of IL-10^+^p-Elk-1^+^ cells in SLE than normal PBMC subsets. These findings imply that Elk-1 in nuclei of SLE PBMCs has been biologically activated likely due to a higher baseline immune activation status, which may enhance ability of Elk-1 to regulate *IL10* transcription. In the absence of microenvironmental activation in control PBMCs, Elk-1 remains within the cytoplasm and less translocated into nuclei, limiting its regulation effects. This hypothesis may in part explain the observation of higher IL-10 expression in SLE patients than healthy controls even if they carry the same risk genotype of rs3122605. In support of this possibility, previous studies revealed an increased expression of activation markers on peripheral lymphocytes of SLE patients, including phosphorylated ERK, JNK and p38 which are prerequisite for subsequent activation of Elk-1 [38], [39]. In addition, our data showed a significantly increased proportion of IL-10^+^p-Elk-1^+^ cells in normal PBMC subsets (Figure 4C) when exposed to IFN-α which may induce a partial activation phenotype in lymphocytes mimicking that of SLE.

We used healthy control, rather than SLE, PBMCs for testing effects of MAPK inhibitors on co-expression of IL-10 and p-Elk-1, because disease activity and medications of SLE patients might confound the results and leucopenia of SLE patients could limit the amount of PBMCs available for our experiments. In all three cell subsets of PBMCs, inhibition of ERK could best suppress IFN-α induced increase in IL-10 and p-Elk-1 co-expression, highlighting the importance for phosphorylated Elk-1 *via* ERK signaling in regulation of *IL10* transcription. The ERK-dependent activation of Elk-1 has been clearly demonstrated in neuronal cells (reviewed in [40]) in which phosphorylation of Elk-1 at S383/389 by ERK is tightly linked to its activation and nuclear translocation and inhibition of phosphorylation results in cytoplasmic Elk-1 retention, limiting its transcriptional properties [22].

Elk-1, like all members of Ets-domain containing transcription factors, can bind genomic regions similarly as well as uniquely to regulate distinct classes of target genes [41]. Another member of the Ets family, Ets-1, has a dual function in regulating *IL10* gene expression acting as both a transcriptional activator with the binding partner Sp-1 in HIV-1Tat-induced *IL10* transcription in THP-1 cells [42], and as a repressor interacting with histone deacetylase 1 (HDAC1) in Th1 cells [43]. Increasing evidence indicates the involvement of Ets-1 in the pathogenesis of SLE: (1) *ETS1* has been identified as a risk locus for SLE in GWAS [44], [45] and the risk allele is associated with decreased levels of *ETS1* transcripts in healthy control PBMCs [45]; (2) Ets-1 is critical in maintaining B cell identity and its absence drives terminal differentiation of B cells into immunoglobulin-secreting plasma cells [46], [47]; (3) Ets-1 functions as a cofactor for T-bet essential for Th1 effector function and differentiation [48]; (4) Ets-1 negatively regulates Th17 cell differentiation and Ets-1 deficiency results in elevated production of IL-17 and IL-17-related cytokines by Th17 cells [49]. These emerging findings support an important role of Ets family transcription factors in the development of SLE manifestations.

Previous findings indicated that increased production of IL-10 by SLE PBMCs was mainly derived from B cells [16], [50]–[53]. One explanation might be due to elevated expression of toll-like receptor 9 (TLR9) on B cells of SLE patients with active disease, as the study showed that TLR9-CpG interaction could enhance the production of anti-dsDNA antibody and IL-10 [54]. B cell receptor (BCR) stimulation or BCR-TLR9 costimulation have been shown to activate the Erk pathway in B cells of NZB×NZW F1 mice [55]. A plausible explanation of elevated co-expression of IL-10 and p-Elk-1 in B cells from SLE patients, especially during active disease, might be attributable to activated ERK and Elk-1 involved in the BCR-dependent IL-10 production in SLE B cells.

Overexpression of B-cell-derived IL-10 contributes to the pathogenesis of SLE likely dependent on its ability to promote B cell proliferation, differentiation and autoantibody production [56], [57]. Recently identified IL-10 producing regulatory B cells (Bregs) may exert immunosuppressive effects to modulate murine lupus [58]–[63]. The phenotypic markers of human Breg cells have not reached consensus; they may be enriched in CD19^+^CD24^hi^CD38^hi^ and CD24^hi^CD27^+^ peripheral blood cells [64], [65]. Interestingly, CD19^+^CD24^hi^CD38^hi^ B cells from SLE patients produce less IL-10 upon stimulation and are functionally impaired in suppressive capacity [64]. Thus, it seems unlikely that Bregs are the major producer of elevated IL-10 we observed in SLE patients.

The minor allele of rs3024505 showed consistently higher frequency in SLE patients than controls in all four ancestries, but only reached statistical significance for association with SLE in EA. Upon considering different genetic models, the additive model yielded the best genotypic association in EA (*P* = 2.7×10^−8^, OR [95%CI] = 1.30 [1.19–1.43]). Given the lack of evidence for genetic heterogeneity across EA, AS, AA and HS (*P* = 0.66 for Q statistic), the lack of significant association between rs3024505 and risk of SLE in AS, AA and HS might be due to low minor allele frequency and small sample size. Under the assumption that the minor allele of rs3024505 confers genetic risk with an odds ratio of 1.3 (determined in EA), the power to detect a significant association (*P*<0.05) for EA samples reaches 100%, whereas it is only 31% in AS, 55% in AA and 58% in HS datasets. According to the 1000 Genome Project data, the LD strength between rs3024505 and our proposed causal SNP rs3122605 is similarly strong in Asians (r^2^ = 1.0) and Americans/Hispanics (r^2^ = 0.8) as in Europeans (r^2^ = 0.85), but not in Africans (r^2^ = 0.1), suggesting that rs3122605 can be tagged by rs3024505 in non-Europeans except for the African-derived population.

It is possible that other SLE-associated variant(s) specific for AS, AA or HS failed to be captured by SNPs used in this study due to different LD pattern in each ancestry. Among non-European populations, SLE GWAS conducted in AA and HS are not in the currently available literature. To our knowledge, there have been four published SLE GWA studies conducted in AS, including two Chinese [44], [45], one Japanese [66] and one Korean GWAS [67], and a meta-analysis study based on Chinese GWAS [68]. In these studies, the Illumina Human 610 BeadChip is the commonly used genotyping platform, which contains nine *IL10* SNPs (including rs3024505) and can capture 23 of the 38 common SNPs (MAF>1%) within +/−5 kb of *IL10* with r^2^>0.9 in Asians (according to the 1000 Genome Project data). Because none of these studies have reported association signals of *IL10* SNPs with SLE, current data are consistent with our findings that the *IL10* locus is not a strong genetic risk factor for SLE in AS. Taken together, the association of *IL10* with SLE in non-EA ancestries awaits further investigation.

We have searched four publically available *cis*-eQTL datasets conducted in immune cells from healthy Europeans [69]–[72] but found no convincing evidence to support the presence of another SNP that can better capture the association signal of *IL10* expression trait in these datasets than our data of SLE-associated rs3024505/rs3122605. In addition, there was no convincing evidence to support the association of rs3024505 with differential expression of other genes within +/−1 Mb flanking region of *IL10*.

Given that the SNP rs3024505 confers increased risk for both SLE and IBD and could be tagged by rs3122605, it is possible that patients with IBD carrying the risk allele of rs3024505 may exhibit high serum levels of IL-10. There is evidence supporting elevated circulating IL-10 levels in both CD and UC patients that positively associated with disease activity [73]–[75], similarly to previous reports in SLE patients. However, genetically engineered mice exhibiting low to no IL-10 signaling in the intestinal tract develop severe IBD manifestations [76]–[78], supporting a pivotal role of IL-10 in down-regulation of inflammation. Increased levels of circulating IL-10 may be elicited by chronic inflammation in IBD, but may not be sufficiently strong to dampen intestinal inflammation [75], raising the possibility of defective IL-10 signaling at sites of organ damage in patients with SLE.

In conclusion, by characterizing genetic variations within the *IL10* gene cluster region, we have identified the *IL10* upstream SNP rs3122605 as the best likely causal variant responsible for association with SLE in European Americans. The SLE-associated rs3122605 G-allele preferentially binds to the activated Elk-1 conferring elevated *IL10* expression. The observation that SLE patients, particular those with increased disease activity, showed enhanced activation of Elk-1 in nuclei and elevated co-expression of IL-10 and phospho-Elk-1 in peripheral lymphocytes highlights the involvement of aberrant Elk-1 signaling in development of SLE and suggests potential targeting therapy for disease amelioration.

## Materials and Methods

### Ethics statement

This study was approved by the Institutional Review Boards (IRBs) or the ethnic committees at the institutions where subjects were recruited. All subjects were enrolled after informed consent had been obtained. The overall study was approved by the IRB of the Oklahoma Medical Research Foundation (OMRF).

### Subjects

To test the association of *IL10* family genes with SLE, we used a large collection of case-control subjects from the collaborative Large Lupus Association Study 2 (LLAS2) [79], including European American (EA: 4,248 cases vs. 3,818 controls), African American (AA: 1,724 vs. 2,024), Asian (AS: 1,328 vs. 1,348) and Hispanic enriched for the Amerindian-European admixture (HS: 1,622 cases vs. 887 controls). African Americans included 286 Gullahs (155 cases vs. 131 controls), who are subjects with African ancestry. Asians were composed primarily of Koreans (906 cases and 1,012 controls) but also included Chinese, Japanese, Taiwanese and Singaporeans. Cases were defined by meeting at least four of the 1997 American College of Rheumatology (ACR) revised criteria for the classification of SLE [80].

To test functional consequences of SLE-associated variants, SLE patients and healthy controls of European ancestry were recruited at the University of California, Los Angeles and through the Lupus Family Registry and Repository (LFRR, lupus.omrf.org) for blood donations.

### Genotyping and quality control

DNA samples were processed at the Lupus Genetics Studies Unit of OMRF. SNP genotyping was performed using an Illumina custom bead array on the iSCAN instrument for 19 tag SNPs covering over 135 kb of *IL10-IL24* region and 347 admixture informative markers (AIMs). SNPs meeting the following criteria were included for subsequent genetic association tests: well-defined cluster scatter plots, SNP call rate >90%, minor allele frequency >1%, total proportion missing <5%, *P*>0.05 for differential missing rate between cases and controls, and Hardy-Weinberg proportion (HWP) test with a *P*>0.01 in controls and *P*>0.0001 in cases.

Subjects with individual genotyping missing rate >10% (due to low quality), shared identity by descent >0.4 or showing mismatch between the reported and estimated gender were removed. The global ancestry of each subject was estimated based on genotype of AIMs, using principal components analysis (PCA) [81] and ADMIXMAP [82]–[84], as described in another LLAS2 study [85], and then genetic outliers were removed.

Finally, a total of unrelated 15,533 subjects including EA (3,820 cases vs. 3,412 controls), AA (1,670 vs. 1,904; composed of 92.5% African Americans and 7.5% Gullahs), AS (1,252 vs. 1,249; composed of 74.6% of Koreans, 16.1% of Chinese and subjects from Japan and Singapore) and HS (1,445 vs. 781) were analyzed for 19 SNPs.

### SNP imputation

Imputation was performed using IMPUTE 2.1.2 [86], with SNP genotypes of 379 Europeans (CEU, TSI, GBR, FIN and IBS), 246 Africans (YRI, ASW and LWK), 286 Asians (CHB, JPT and CHS) and 181 Americans (MXL, PUR and CLM) from the 1000 Genomes Project (version 3 of the phase 1 integrated data, March 2012 release) as references in imputation for our EA, AA, AS and HS subjects, respectively. Imputed genotypes had to meet the threshold of information score >0.9, as well as the quality control criteria as described above. After imputation, we obtained an additional 109 SNPs for EA, 45 for AA, 80 for AS and 64 for HS (the number varied due to different LD structure) for further analysis.

### Real-time quantitative PCR

Total RNA was purified with TRIzol reagent (Life Technologies) from PBMCs of EA individuals (58 SLE cases and 55 healthy controls) and reverse-transcribed into cDNA with SuperScript II Reverse Transcriptase kit (Life Technologies). Messenger RNA levels of *IL10* and a housekeeping gene *RPLP0* were measured by quantitative real-time PCR using TaqMan assays (*IL10* probe: Hs00961622_m1; *RPLP0* probe: Hs99999902_m1, Applied Biosystems). All samples were run in triplicate. Relative *IL10* mRNA levels were normalized to that of *RPLP0*, calculated by the 2^−ΔΔCt^ method and Log10 transformed.

### Enzyme-linked immunosorbent assay (ELISA)

Plasma IL-10 levels from 132 SLE patients and 116 healthy controls of EA ancestry were measured by ELISA (R&D systems).

### Cell cultures

To examine whether inhibition of MAPK pathway may affect co-expression of IL-10 and p-Elk-1, control PBMCs (1×10^6^) were cultured in growth medium with or without interferon alpha (IFNα) (1000 U/ml; PBL Biomedical Laboratories) in the presence or absence of one MAPK inhibitor (EMD Millipore), PD 98059 (20 µM; ERK/MEK inhibitor), SP 600125 (20 µM; JNK inhibitor) or SB 203580 (10 µM; p38-MAPK pathway inhibitor), respectively. Addition of Brefeldin A (eBiosciense) to cells in culture blocks intracellular protein (IL-10) transport processes.

### Flow cytometry

The patients with SLE recruited in this part of study were evaluated for disease activity by the SLE Disease Activity Index (SLEDAI) 2000 [87] at the time of blood draw, and SLEDAI≥4 was considered as active disease [20], [21]. Freshly isolated or cultured PBMCs were incubated with mouse reference serum to block nonspecific binding to Fcγ receptors and then incubated with peridinin chlorophyll protein (PerCP)-conjugated anti-human CD3, allophycocyanin (APC)-conjugated anti-human CD19 and phycoerythrin (PE)-conjugated anti-human CD14 (eBiosciense) to identify T cell, B cell and monocyte subpopulations, respectively. For intracellular staining of IL-10 and p-Elk-1, cells were fixed with IC Fixation Buffer (eBiosciense), washed with Permeabilization Buffer (eBiosciense), and stained with fluorescein isothiocyanate (FITC)-conjugated anti-human IL-10 (eBiosciense) and PE- or Alexa Fluor647-conjugated anti-phospho-Elk-1 antibody (BD Biosciences). Background fluorescence was assessed using appropriate isotype- and fluorochrome-matched control antibodies. Cells were collected and analyzed by FACSCalibur flow cytometer equipped with the manufacturer's software (CellQuest; BD Biosciences).

### Bioinformatic prediction of transcription factors, electrophoretic mobility shift assay (EMSA) and supershift assay

Bioinformatic analysis using the program TFSEARCH (conducted on 01/18/2011) showed predicted binding to Elk-1 and STRE in DNA sequence containing the minor but not the major allele of rs3122605 (Figure S3 and S4). In addition, HSF, ADR1 and MZF1 were predicted to bind with sequence containing either allele of rs3122605. Given that we were interested in identifying transcription factors that preferentially bind to the minor allele of rs3122605, and that the STRE (stress response element) binding factor includes two yeast transcription factors, Msn2p and Msn4p, we prioritized to test Elk-1 using EMSA.

EMSA and supershift assays were performed as previously described [88]. Nuclear extracts were prepared from peripheral blood lymphocytes of SLE patients with active disease using NE-PER Nuclear Extraction Reagent (Thermo scientific) and incubated with biotin-labeled oligodeoxynucleotides (synthesized by Integrated DNA Technologies, depicted in Table S3). EMSAs were performed with the LightShift Chemiluminescent EMSA kit (Thermo scientific). The antibody used in the supershift reactions was polyclonal rabbit anti-human Elk-1 (Santa Cruz Biotechnology).

### Immunoblot analyses

Cytoplasmic or nuclear proteins from PBMCs were prepared using NE-PER Nuclear Extraction Reagent (Thermo scientific). Following SDS/PAGE, proteins were transferred onto Immobilon-P membrane (Millipore). After blocking with membrane blocking solution (Invitrogen, Life Technologies), the membrane was successively incubated with the anti-Elk-1 (Santa Cruz Biotechnology) or anti-phospho-Elk-1 (Cell Signaling Technology) primary antibody and the horseradish peroxidase (HRP)-conjugated secondary antibody (Santa Cruz Biotechnology). Blots were developed using the ECLPlus Western Blotting Detection System (GE Healthcare), visualized with ChemiDoc XRS imager and analyzed by Quantity One software (BIO-RAD). β-actin or Lamin B was used as internal control.

### Statistical analysis

Allelic association tests in each ancestral group and conditional haplotype-based association tests in EA ancestry were performed by PLINK v1.07 software [89] under a logistic regression model adjusted for gender and the first three principal components estimated using AIMs. The Bonferroni corrected *P*-value threshold was adjusted to *P*<3.9×10^−4^ ( = 0.05/128 SNPs in EA). Pairwised LD values between SNPs and haplotypic association with SLE were evaluated using Haploview 4.2 [90]. The linear regression test was used to evaluate the association of SNP genotypes with IL-10 mRNA or protein levels. The Student's *t*-test was used to compare the mean values between two groups. A *P* value<0.05 was considered to be statistically significant.

## Supporting Information

Figure S1 No nuclear protein bindings conferred by rs3024505, rs3024493, rs3024495 and rs61815643. In EMSA, oligodeoxynucleotide probes containing the risk and non-risk alleles of rs3024505 (A), rs3024493 (B), rs3024495 (C) and rs61815643 (D) were incubated with nuclear extracts of peripheral blood lymphocytes from active SLE patients. Competition analysis using excess amounts of unlabeled self-competitor confirmed that shift bands produced by probes of rs3024493 and rs61815643 were not specific (N.S.). The data are representative of two independent experiments.
(TIF)

Figure S2 IL-10 expression in T cells, B cells and monocytes. Representative contour plot and quantification of IL-10-producing CD3^+^ T cells, CD19^+^ B cells and CD14^+^ monocytes in (A) normal PBMCs treated with or without IFNα for 24 hours, and in (B) PBMCs from patients with SLE. (C) CD19-gated PBMC population was used for the purity of B cells. The gate indicates the percentage of IL-10 producing B cells from patients with SLE. Data are represented as mean ± SD percentage of positive cells obtained in three independent experiments using different individuals.
(TIF)

Figure S3 TFSEARCH search result of the SLE-risk minor allele of rs3122605.
(TIF)

Figure S4 TFSEARCH search result of the major allele of rs3122605.
(TIF)

Table S1 Association of *IL10* SNPs with SLE in European Americans. Position of each SNP is based on GRch37/hg19. Only SNPs with *P*<0.05 were tested in conditional testing. Four SLE-associated *IL10* SNPs are highlighted in bold. Abbreviation: G, genotyped; I, imputed; ND, not distinguished; OR, odds ratio; -, missing data.
(DOC)

Table S2 Association of *IL10* cluster SNPs with SLE in Non-European ancestral groups. Position of each SNP is based on GRch37/hg19. Missing data in SNP imputation is denoted as ‘–’. Four SLE-associated SNPs identified in European Americans are highlighted in bold. Abbreviation: G, genotyped; I, imputed; OR, odds ratio.
(DOC)

Table S3 DNA sequences of oligodeoxynucleotide probes used in EMSA.
(DOC)

## References

[1] Johanneson B, Lima G, von Salome J, Alarcon-Segovia D, Alarcon-Riquelme ME (2002) A major susceptibility locus for systemic lupus erythemathosus maps to chromosome 1q31. Am J Hum Genet 71: 1060–1071. 12373647 10.1086/344289 PMC385085

[2] Wu H, Boackle SA, Hanvivadhanakul P, Ulgiati D, Grossman JM, et al (2007) Association of a common complement receptor 2 haplotype with increased risk of systemic lupus erythematosus. Proc Natl Acad Sci U S A 104: 3961–3966. 17360460 10.1073/pnas.0609101104 PMC1820691

[3] Gateva V, Sandling JK, Hom G, Taylor KE, Chung SA, et al (2009) A large-scale replication study identifies TNIP1, PRDM1, JAZF1, UHRF1BP1 and IL10 as risk loci for systemic lupus erythematosus. Nat Genet 41: 1228–1233. 19838195 10.1038/ng.468 PMC2925843

[4] Franke A, McGovern DP, Barrett JC, Wang K, Radford-Smith GL, et al (2010) Genome-wide meta-analysis increases to 71 the number of confirmed Crohn's disease susceptibility loci. Nat Genet 42: 1118–1125. 21102463 10.1038/ng.717 PMC3299551

[5] Franke A, Balschun T, Karlsen TH, Sventoraityte J, Nikolaus S, et al (2008) Sequence variants in IL10, ARPC2 and multiple other loci contribute to ulcerative colitis susceptibility. Nat Genet 40: 1319–1323. 18836448 10.1038/ng.221

[6] Anderson CA, Boucher G, Lees CW, Franke A, D'Amato M, et al (2011) Meta-analysis identifies 29 additional ulcerative colitis risk loci, increasing the number of confirmed associations to 47. Nat Genet 43: 246–252. 21297633 10.1038/ng.764 PMC3084597

[7] Barrett JC, Clayton DG, Concannon P, Akolkar B, Cooper JD, et al (2009) Genome-wide association study and meta-analysis find that over 40 loci affect risk of type 1 diabetes. Nat Genet 41: 703–707. 19430480 10.1038/ng.381 PMC2889014

[8] Plagnol V, Howson JM, Smyth DJ, Walker N, Hafler JP, et al (2011) Genome-wide association analysis of autoantibody positivity in type 1 diabetes cases. PLoS Genet 7: e1002216. 21829393 10.1371/journal.pgen.1002216 PMC3150451

[9] Remmers EF, Cosan F, Kirino Y, Ombrello MJ, Abaci N, et al (2010) Genome-wide association study identifies variants in the MHC class I, IL10, and IL23R-IL12RB2 regions associated with Behcet's disease. Nat Genet 42: 698–702. 20622878 10.1038/ng.625 PMC2923807

[10] Mizuki N, Meguro A, Ota M, Ohno S, Shiota T, et al (2010) Genome-wide association studies identify IL23R-IL12RB2 and IL10 as Behcet's disease susceptibility loci. Nat Genet 42: 703–706. 20622879 10.1038/ng.624

[11] Hofmann SR, Rosen-Wolff A, Tsokos GC, Hedrich CM (2012) Biological properties and regulation of IL-10 related cytokines and their contribution to autoimmune disease and tissue injury. Clin Immunol 143: 116–127. 22459704 10.1016/j.clim.2012.02.005

[12] Pestka S, Krause CD, Sarkar D, Walter MR, Shi Y, et al (2004) Interleukin-10 and related cytokines and receptors. Annu Rev Immunol 22: 929–979. 15032600 10.1146/annurev.immunol.22.012703.104622

[13] Park YB, Lee SK, Kim DS, Lee J, Lee CH, et al (1998) Elevated interleukin-10 levels correlated with disease activity in systemic lupus erythematosus. Clin Exp Rheumatol 16: 283–288. 9631750

[14] Houssiau FA, Lefebvre C, Vanden Berghe M, Lambert M, Devogelaer JP, et al (1995) Serum interleukin 10 titers in systemic lupus erythematosus reflect disease activity. Lupus 4: 393–395. 8563734 10.1177/096120339500400510

[15] Llorente L, Richaud-Patin Y, Garcia-Padilla C, Claret E, Jakez-Ocampo J, et al (2000) Clinical and biologic effects of anti-interleukin-10 monoclonal antibody administration in systemic lupus erythematosus. Arthritis Rheum 43: 1790–1800. 10943869 10.1002/1529-0131(200008)43:8<1790::AID-ANR15>3.0.CO;2-2

[16] Llorente L, Richaud-Patin Y, Couderc J, Alarcon-Segovia D, Ruiz-Soto R, et al (1997) Dysregulation of interleukin-10 production in relatives of patients with systemic lupus erythematosus. Arthritis Rheum 40: 1429–1435. 9259422 10.1002/art.1780400810

[17] Grondal G, Kristjansdottir H, Gunnlaugsdottir B, Arnason A, Lundberg I, et al (1999) Increased number of interleukin-10-producing cells in systemic lupus erythematosus patients and their first-degree relatives and spouses in Icelandic multicase families. Arthritis Rheum 42: 1649–1654. 10446864 10.1002/1529-0131(199908)42:8<1649::AID-ANR13>3.0.CO;2-D

[18] Beebe AM, Cua DJ, de Waal Malefyt R (2002) The role of interleukin-10 in autoimmune disease: systemic lupus erythematosus (SLE) and multiple sclerosis (MS). Cytokine Growth Factor Rev 13: 403–412. 12220553 10.1016/s1359-6101(02)00025-4

[19] Okamoto A, Fujio K, Okamura T, Yamamoto K (2011) Regulatory T-cell-associated cytokines in systemic lupus erythematosus. J Biomed Biotechnol 2011: 463412. 22219657 10.1155/2011/463412 PMC3247013

[20] Gladman DD, Urowitz MB, Kagal A, Hallett D (2000) Accurately describing changes in disease activity in Systemic Lupus Erythematosus. J Rheumatol 27: 377–379. 10685800

[21] Yee CS, Farewell VT, Isenberg DA, Griffiths B, Teh LS, et al (2011) The use of Systemic Lupus Erythematosus Disease Activity Index-2000 to define active disease and minimal clinically meaningful change based on data from a large cohort of systemic lupus erythematosus patients. Rheumatology (Oxford) 50: 982–988. 21245073 10.1093/rheumatology/keq376 PMC3077910

[22] Lavaur J, Bernard F, Trifilieff P, Pascoli V, Kappes V, et al (2007) A TAT-DEF-Elk-1 peptide regulates the cytonuclear trafficking of Elk-1 and controls cytoskeleton dynamics. J Neurosci 27: 14448–14458. 18160653 10.1523/JNEUROSCI.2279-07.2007 PMC6673434

[23] Banchereau J, Pascual V (2006) Type I interferon in systemic lupus erythematosus and other autoimmune diseases. Immunity 25: 383–392. 16979570 10.1016/j.immuni.2006.08.010

[24] van der Linden MW, Westendorp RG, Sturk A, Bergman W, Huizinga TW (2000) High interleukin-10 production in first-degree relatives of patients with generalized but not cutaneous lupus erythematosus. J Investig Med 48: 327–334. 10979237

[25] Grondal G, Kristjansdottir H, Gunnlaugsdottir B, Arnason A, Lundberg I, et al (1999) Increased number of interleukin-10-producing cells in systemic lupus erythematosus patients and their first-degree relatives and spouses in Icelandic multicase families. Arthritis Rheum 42: 1649–1654. 10446864 10.1002/1529-0131(199908)42:8<1649::AID-ANR13>3.0.CO;2-D

[26] Eskdale J, Wordsworth P, Bowman S, Field M, Gallagher G (1997) Association between polymorphisms at the human IL-10 locus and systemic lupus erythematosus. Tissue Antigens 49: 635–639. 9234486 10.1111/j.1399-0039.1997.tb02812.x

[27] Mehrian R, Quismorio FP Jr, Strassmann G, Stimmler MM, Horwitz DA, et al (1998) Synergistic effect between IL-10 and bcl-2 genotypes in determining susceptibility to systemic lupus erythematosus. Arthritis Rheum 41: 596–602. 9550468 10.1002/1529-0131(199804)41:4<596::AID-ART6>3.0.CO;2-2

[28] D'Alfonso S, Rampi M, Bocchio D, Colombo G, Scorza-Smeraldi R, et al (2000) Systemic lupus erythematosus candidate genes in the Italian population: evidence for a significant association with interleukin-10. Arthritis Rheum 43: 120–128. 10643707 10.1002/1529-0131(200001)43:1<120::AID-ANR15>3.0.CO;2-3

[29] Mok CC, Lanchbury JS, Chan DW, Lau CS (1998) Interleukin-10 promoter polymorphisms in Southern Chinese patients with systemic lupus erythematosus. Arthritis Rheum 41: 1090–1095. 9627019 10.1002/1529-0131(199806)41:6<1090::AID-ART16>3.0.CO;2-6

[30] Rood MJ, Keijsers V, van der Linden MW, Tong TQ, Borggreve SE, et al (1999) Neuropsychiatric systemic lupus erythematosus is associated with imbalance in interleukin 10 promoter haplotypes. Ann Rheum Dis 58: 85–89. 10343522 10.1136/ard.58.2.85 PMC1752835

[31] Gibson AW, Edberg JC, Wu J, Westendorp RG, Huizinga TW, et al (2001) Novel single nucleotide polymorphisms in the distal IL-10 promoter affect IL-10 production and enhance the risk of systemic lupus erythematosus. J Immunol 166: 3915–3922. 11238636 10.4049/jimmunol.166.6.3915

[32] Trynka G, Sandor C, Han B, Xu H, Stranger BE, et al (2013) Chromatin marks identify critical cell types for fine mapping complex trait variants. Nat Genet 45: 124–130. 23263488 10.1038/ng.2504 PMC3826950

[33] Sharrocks AD (2001) The ETS-domain transcription factor family. Nat Rev Mol Cell Biol 2: 827–837. 11715049 10.1038/35099076

[34] Rao VN, Reddy ES (1994) elk-1 proteins interact with MAP kinases. Oncogene 9: 1855–1860. 8208531

[35] Li W, Marshall C, Mei L, Dzubow L, Schmults C, et al (2005) Srcasm modulates EGF and Src-kinase signaling in keratinocytes. J Biol Chem 280: 6036–6046. 15579470 10.1074/jbc.M406546200

[36] Sharma A, Callahan LM, Sul JY, Kim TK, Barrett L, et al (2010) A neurotoxic phosphoform of Elk-1 associates with inclusions from multiple neurodegenerative diseases. PLoS One 5: e9002. 20126313 10.1371/journal.pone.0009002 PMC2814869

[37] Morris JF, Sul JY, Kim MS, Klein-Szanto AJ, Schochet T, et al (2013) Elk-1 phosphorylated at threonine-417 is present in diverse cancers and correlates with differentiation grade of colonic adenocarcinoma. Hum Pathol 44: 766–776. 23114923 10.1016/j.humpath.2012.08.001 PMC4487418

[38] Grammer AC, Fischer R, Lee O, Zhang X, Lipsky PE (2004) Flow cytometric assessment of the signaling status of human B lymphocytes from normal and autoimmune individuals. Arthritis Res Ther 6: 28–38. 14979930 10.1186/ar1155 PMC400425

[39] Wong CK, Wong PT, Tam LS, Li EK, Chen DP, et al (2009) Activation profile of intracellular mitogen-activated protein kinases in peripheral lymphocytes of patients with systemic lupus erythematosus. J Clin Immunol 29: 738–746. 19756990 10.1007/s10875-009-9318-4

[40] Besnard A, Galan-Rodriguez B, Vanhoutte P, Caboche J (2011) Elk-1 a transcription factor with multiple facets in the brain. Front Neurosci 5: 35. 21441990 10.3389/fnins.2011.00035 PMC3060702

[41] Odrowaz Z, Sharrocks AD (2012) ELK1 uses different DNA binding modes to regulate functionally distinct classes of target genes. PLoS Genet 8: e1002694. 22589737 10.1371/journal.pgen.1002694 PMC3349735

[42] Li JC, Lau AS (2007) A role for mitogen-activated protein kinase and Ets-1 in the induction of interleukin-10 transcription by human immunodeficiency virus-1 Tat. Immunology 121: 337–348. 17376198 10.1111/j.1365-2567.2007.02580.x PMC2265950

[43] Lee CG, Kwon HK, Sahoo A, Hwang W, So JS, et al (2012) Interaction of Ets-1 with HDAC1 represses IL-10 expression in Th1 cells. J Immunol 188: 2244–2253. 22266280 10.4049/jimmunol.1101614

[44] Han JW, Zheng HF, Cui Y, Sun LD, Ye DQ, et al (2009) Genome-wide association study in a Chinese Han population identifies nine new susceptibility loci for systemic lupus erythematosus. Nat Genet 41: 1234–1237. 19838193 10.1038/ng.472

[45] Yang W, Shen N, Ye DQ, Liu Q, Zhang Y, et al (2010) Genome-Wide Association Study in Asian Populations Identifies Variants in ETS1 and WDFY4 Associated with Systemic Lupus Erythematosus. PLoS Genet 6: e1000841. 20169177 10.1371/journal.pgen.1000841 PMC2820522

[46] Eyquem S, Chemin K, Fasseu M, Chopin M, Sigaux F, et al (2004) The development of early and mature B cells is impaired in mice deficient for the Ets-1 transcription factor. Eur J Immunol 34: 3187–3196. 15384043 10.1002/eji.200425352

[47] Wang D, John SA, Clements JL, Percy DH, Barton KP, et al (2005) Ets-1 deficiency leads to altered B cell differentiation, hyperresponsiveness to TLR9 and autoimmune disease. Int Immunol 17: 1179–1191. 16051621 10.1093/intimm/dxh295

[48] Grenningloh R, Kang BY, Ho IC (2005) Ets-1, a functional cofactor of T-bet, is essential for Th1 inflammatory responses. J Exp Med 201: 615–626. 15728239 10.1084/jem.20041330 PMC2213045

[49] Moisan J, Grenningloh R, Bettelli E, Oukka M, Ho IC (2007) Ets-1 is a negative regulator of Th17 differentiation. J Exp Med 204: 2825–2835. 17967903 10.1084/jem.20070994 PMC2118518

[50] Llorente L, Richaud-Patin Y, Wijdenes J, Alcocer-Varela J, Maillot MC, et al (1993) Spontaneous production of interleukin-10 by B lymphocytes and monocytes in systemic lupus erythematosus. Eur Cytokine Netw 4: 421–427. 8186374

[51] Llorente L, Richaud-Patin Y, Fior R, Alcocer-Varela J, Wijdenes J, et al (1994) In vivo production of interleukin-10 by non-T cells in rheumatoid arthritis, Sjogren's syndrome, and systemic lupus erythematosus. A potential mechanism of B lymphocyte hyperactivity and autoimmunity. Arthritis Rheum 37: 1647–1655. 7980676 10.1002/art.1780371114

[52] al-Janadi M, al-Dalaan A, al-Balla S, al-Humaidi M, Raziuddin S (1996) Interleukin-10 (IL-10) secretion in systemic lupus erythematosus and rheumatoid arthritis: IL-10-dependent CD4+CD45RO+ T cell-B cell antibody synthesis. J Clin Immunol 16: 198–207. 8840221 10.1007/BF01541225

[53] Csiszar A, Nagy G, Gergely P, Pozsonyi T, Pocsik E (2000) Increased interferon-gamma (IFN-gamma), IL-10 and decreased IL-4 mRNA expression in peripheral blood mononuclear cells (PBMC) from patients with systemic lupus erythematosus (SLE). Clin Exp Immunol 122: 464–470. 11122256 10.1046/j.1365-2249.2000.01369.x PMC1905797

[54] Nakano S, Morimoto S, Suzuki J, Nozawa K, Amano H, et al (2008) Role of pathogenic auto-antibody production by Toll-like receptor 9 of B cells in active systemic lupus erythematosus. Rheumatology (Oxford) 47: 145–149. 18160420 10.1093/rheumatology/kem327

[55] Schickel JN, Pasquali JL, Soley A, Knapp AM, Decossas M, et al (2012) Carabin deficiency in B cells increases BCR-TLR9 costimulation-induced autoimmunity. EMBO Mol Med 4: 1261–1275. 23109291 10.1002/emmm.201201595 PMC3531602

[56] Rousset F, Garcia E, Defrance T, Peronne C, Vezzio N, et al (1992) Interleukin 10 is a potent growth and differentiation factor for activated human B lymphocytes. Proc Natl Acad Sci U S A 89: 1890–1893. 1371884 10.1073/pnas.89.5.1890 PMC48559

[57] Llorente L, Richaud-Patin Y (2003) The role of interleukin-10 in systemic lupus erythematosus. J Autoimmun 20: 287–289. 12791314 10.1016/s0896-8411(03)00043-x

[58] Yang M, Rui K, Wang S, Lu L (2013) Regulatory B cells in autoimmune diseases. Cell Mol Immunol 10: 122–132. 23292280 10.1038/cmi.2012.60 PMC4003045

[59] Blair PA, Chavez-Rueda KA, Evans JG, Shlomchik MJ, Eddaoudi A, et al (2009) Selective targeting of B cells with agonistic anti-CD40 is an efficacious strategy for the generation of induced regulatory T2-like B cells and for the suppression of lupus in MRL/lpr mice. J Immunol 182: 3492–3502. 19265127 10.4049/jimmunol.0803052 PMC4082659

[60] Haas KM, Watanabe R, Matsushita T, Nakashima H, Ishiura N, et al (2010) Protective and pathogenic roles for B cells during systemic autoimmunity in NZB/W F1 mice. J Immunol 184: 4789–4800. 20368280 10.4049/jimmunol.0902391 PMC3734557

[61] Watanabe R, Ishiura N, Nakashima H, Kuwano Y, Okochi H, et al (2010) Regulatory B cells (B10 cells) have a suppressive role in murine lupus: CD19 and B10 cell deficiency exacerbates systemic autoimmunity. J Immunol 184: 4801–4809. 20368271 10.4049/jimmunol.0902385 PMC3734559

[62] Scapini P, Lamagna C, Hu Y, Lee K, Tang Q, et al (2011) B cell-derived IL-10 suppresses inflammatory disease in Lyn-deficient mice. Proc Natl Acad Sci U S A 108: E823–832. 21911371 10.1073/pnas.1107913108 PMC3193193

[63] Teichmann LL, Kashgarian M, Weaver CT, Roers A, Muller W, et al (2012) B cell-derived IL-10 does not regulate spontaneous systemic autoimmunity in MRL.Fas(lpr) mice. J Immunol 188: 678–685. 22156495 10.4049/jimmunol.1102456 PMC3253138

[64] Blair PA, Norena LY, Flores-Borja F, Rawlings DJ, Isenberg DA, et al (2010) CD19(+)CD24(hi)CD38(hi) B cells exhibit regulatory capacity in healthy individuals but are functionally impaired in systemic Lupus Erythematosus patients. Immunity 32: 129–140. 20079667 10.1016/j.immuni.2009.11.009

[65] Iwata Y, Matsushita T, Horikawa M, Dilillo DJ, Yanaba K, et al (2011) Characterization of a rare IL-10-competent B-cell subset in humans that parallels mouse regulatory B10 cells. Blood 117: 530–541. 20962324 10.1182/blood-2010-07-294249 PMC3031478

[66] Okada Y, Shimane K, Kochi Y, Tahira T, Suzuki A, et al (2012) A genome-wide association study identified AFF1 as a susceptibility locus for systemic lupus eyrthematosus in Japanese. PLoS Genet 8: e1002455. 22291604 10.1371/journal.pgen.1002455 PMC3266877

[67] Lee HS, Kim T, Bang SY, Na YJ, Kim I, et al (2013) Ethnic specificity of lupus-associated loci identified in a genome-wide association study in Korean women. Ann Rheum Dis [Epub ahead of print]. 10.1136/annrheumdis-2012-202675 23740238

[68] Yang W, Tang H, Zhang Y, Tang X, Zhang J, et al (2013) Meta-analysis followed by replication identifies loci in or near CDKN1B, TET3, CD80, DRAM1, and ARID5B as associated with systemic lupus erythematosus in Asians. Am J Hum Genet 92: 41–51. 23273568 10.1016/j.ajhg.2012.11.018 PMC3542470

[69] Fairfax BP, Makino S, Radhakrishnan J, Plant K, Leslie S, et al (2012) Genetics of gene expression in primary immune cells identifies cell type-specific master regulators and roles of HLA alleles. Nat Genet 44: 502–510. 22446964 10.1038/ng.2205 PMC3437404

[70] Grundberg E, Small KS, Hedman AK, Nica AC, Buil A, et al (2012) Mapping cis- and trans-regulatory effects across multiple tissues in twins. Nat Genet 44: 1084–1089. 22941192 10.1038/ng.2394 PMC3784328

[71] Dimas AS, Deutsch S, Stranger BE, Montgomery SB, Borel C, et al (2009) Common regulatory variation impacts gene expression in a cell type-dependent manner. Science 325: 1246–1250. 19644074 10.1126/science.1174148 PMC2867218

[72] Stranger BE, Montgomery SB, Dimas AS, Parts L, Stegle O, et al (2012) Patterns of cis regulatory variation in diverse human populations. PLoS Genet 8: e1002639. 22532805 10.1371/journal.pgen.1002639 PMC3330104

[73] Szkaradkiewicz A, Marciniak R, Chudzicka-Strugala I, Wasilewska A, Drews M, et al (2009) Proinflammatory cytokines and IL-10 in inflammatory bowel disease and colorectal cancer patients. Arch Immunol Ther Exp (Warsz) 57: 291–294. 19578817 10.1007/s00005-009-0031-z

[74] Wang AH, Lam WJ, Han DY, Ding Y, Hu R, et al (2011) The effect of IL-10 genetic variation and interleukin 10 serum levels on Crohn's disease susceptibility in a New Zealand population. Hum Immunol 72: 431–435. 21354456 10.1016/j.humimm.2011.02.014

[75] Kucharzik T, Stoll R, Lugering N, Domschke W (1995) Circulating antiinflammatory cytokine IL-10 in patients with inflammatory bowel disease (IBD). Clin Exp Immunol 100: 452–456. 7774055 10.1111/j.1365-2249.1995.tb03721.x PMC1534463

[76] Kuhn R, Lohler J, Rennick D, Rajewsky K, Muller W (1993) Interleukin-10-deficient mice develop chronic enterocolitis. Cell 75: 263–274. 8402911 10.1016/0092-8674(93)80068-p

[77] Spencer SD, Di Marco F, Hooley J, Pitts-Meek S, Bauer M, et al (1998) The orphan receptor CRF2-4 is an essential subunit of the interleukin 10 receptor. J Exp Med 187: 571–578. 9463407 10.1084/jem.187.4.571 PMC2212143

[78] Glocker EO, Kotlarz D, Boztug K, Gertz EM, Schaffer AA, et al (2009) Inflammatory bowel disease and mutations affecting the interleukin-10 receptor. N Engl J Med 361: 2033–2045. 19890111 10.1056/NEJMoa0907206 PMC2787406

[79] Scofield RH, Kaufman KM (2012) Mapping susceptibility gene in systemic lupus erythematosus. Methods Mol Biol 900: 11–24. 22933063 10.1007/978-1-60761-720-4_2

[80] Hochberg MC (1997) Updating the American College of Rheumatology revised criteria for the classification of systemic lupus erythematosus. Arthritis Rheum 40: 1725. 9324032 10.1002/art.1780400928

[81] Price AL, Patterson NJ, Plenge RM, Weinblatt ME, Shadick NA, et al (2006) Principal components analysis corrects for stratification in genome-wide association studies. Nat Genet 38: 904–909. 16862161 10.1038/ng1847

[82] Hoggart CJ, Parra EJ, Shriver MD, Bonilla C, Kittles RA, et al (2003) Control of confounding of genetic associations in stratified populations. Am J Hum Genet 72: 1492–1504. 12817591 10.1086/375613 PMC1180309

[83] Hoggart CJ, Shriver MD, Kittles RA, Clayton DG, McKeigue PM (2004) Design and analysis of admixture mapping studies. Am J Hum Genet 74: 965–978. 15088268 10.1086/420855 PMC1181989

[84] McKeigue PM (2005) Prospects for admixture mapping of complex traits. Am J Hum Genet 76: 1–7. 15540159 10.1086/426949 PMC1196412

[85] Lessard CJ, Adrianto I, Kelly JA, Kaufman KM, Grundahl KM, et al (2011) Identification of a systemic lupus erythematosus susceptibility locus at 11p13 between PDHX and CD44 in a multiethnic study. Am J Hum Genet 88: 83–91. 21194677 10.1016/j.ajhg.2010.11.014 PMC3014359

[86] Howie BN, Donnelly P, Marchini J (2009) A flexible and accurate genotype imputation method for the next generation of genome-wide association studies. PLoS Genet 5: e1000529. 19543373 10.1371/journal.pgen.1000529 PMC2689936

[87] Gladman DD, Ibanez D, Urowitz MB (2002) Systemic lupus erythematosus disease activity index 2000. J Rheumatol 29: 288–291. 11838846

[88] Tan W, Wu H, Zhao J, Derber LA, Lee DM, et al (2010) A functional RANKL polymorphism associated with younger age at onset of rheumatoid arthritis. Arthritis Rheum 62: 2864–2875. 20533289 10.1002/art.27589 PMC2944013

[89] Purcell S, Neale B, Todd-Brown K, Thomas L, Ferreira MA, et al (2007) PLINK: a tool set for whole-genome association and population-based linkage analyses. Am J Hum Genet 81: 559–575. 17701901 10.1086/519795 PMC1950838

[90] Barrett JC, Fry B, Maller J, Daly MJ (2005) Haploview: analysis and visualization of LD and haplotype maps. Bioinformatics 21: 263–265. 15297300 10.1093/bioinformatics/bth457

