# Supplementary figures and images for: Preferential Binding to Elk-1 by SLE-Associated IL10 Risk Allele Upregulates IL10 Expression
Source: PLoS Genet. 2013 Oct 10;9(10):e1003870. doi: 10.1371/journal.pgen.1003870 (PMC3794920; doi:10.1371/journal.pgen.1003870)

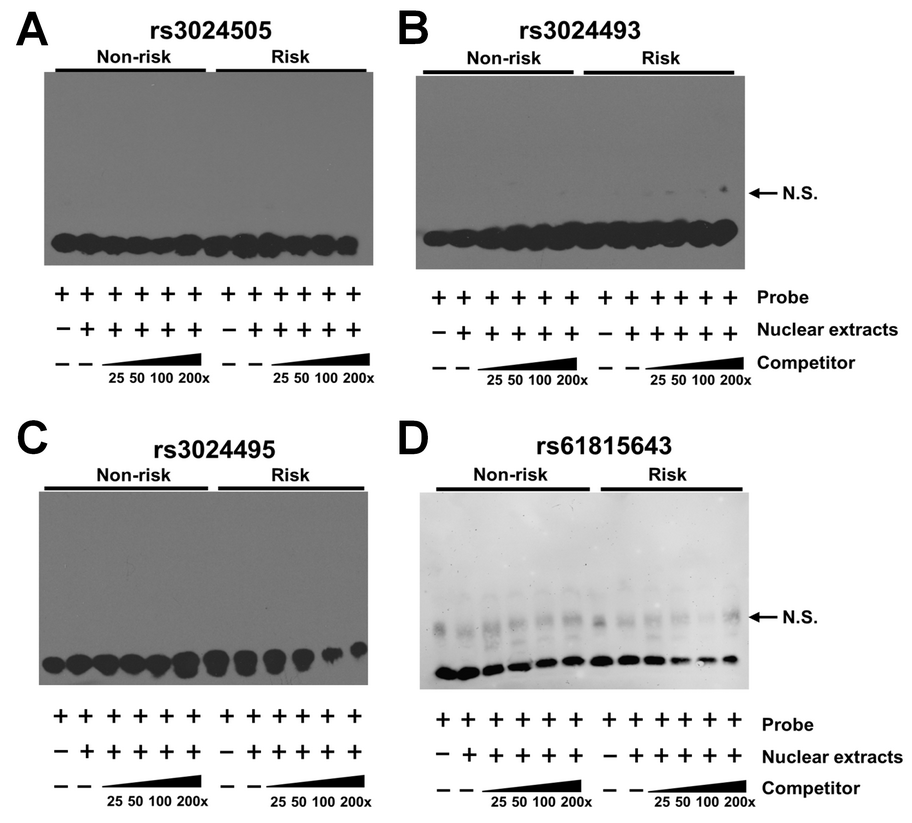

Supplement: Figure S1 — No nuclear protein bindings conferred by rs3024505, rs3024493, rs3024495 and rs61815643. In EMSA, oligodeoxynucleotide probes containing the risk and non-risk alleles of rs3024505 (A), rs3024493 (B), rs3024495 (C) and rs61815643 (D) were incubated with nuclear extracts of peripheral blood lymphocytes from active SLE patients. Competition analysis using excess amounts of unlabeled self-competitor confirmed that shift bands produced by probes of rs3024493 and rs61815643 were not specific (N.S.). The data are representative of two independent experiments. (TIF) [file pgen.1003870.s001.tif]

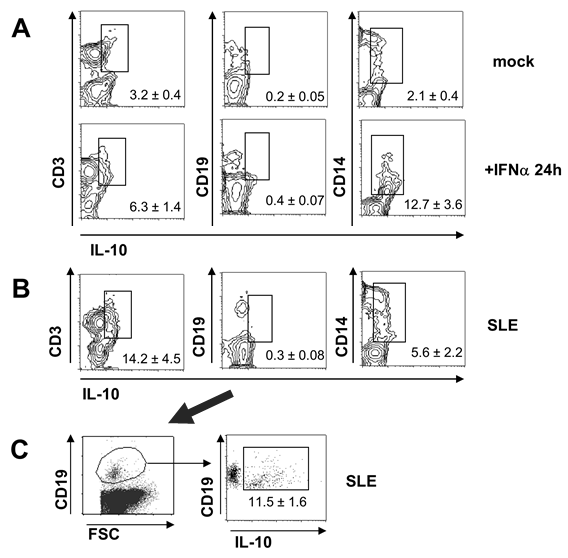

Supplement: Figure S2 — IL-10 expression in T cells, B cells and monocytes. Representative contour plot and quantification of IL-10-producing CD3+ T cells, CD19+ B cells and CD14+ monocytes in (A) normal PBMCs treated with or without IFNα for 24 hours, and in (B) PBMCs from patients with SLE. (C) CD19-gated PBMC population was used for the purity of B cells. The gate indicates the percentage of IL-10 producing B cells from patients with SLE. Data are represented as mean ± SD percentage of positive cells obtained in three independent experiments using different individuals. (TIF) [file pgen.1003870.s002.tif]

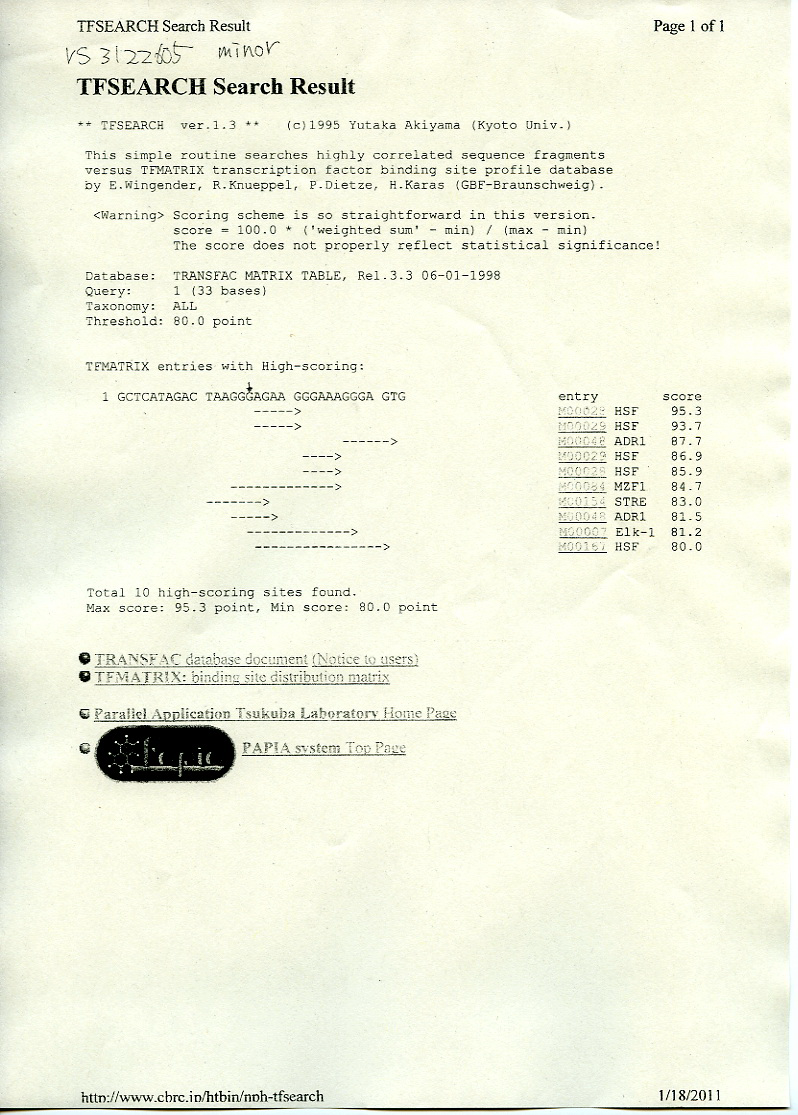

Supplement: Figure S3 — TFSEARCH search result of the SLE-risk minor allele of rs3122605. (TIF) [file pgen.1003870.s003.tif]

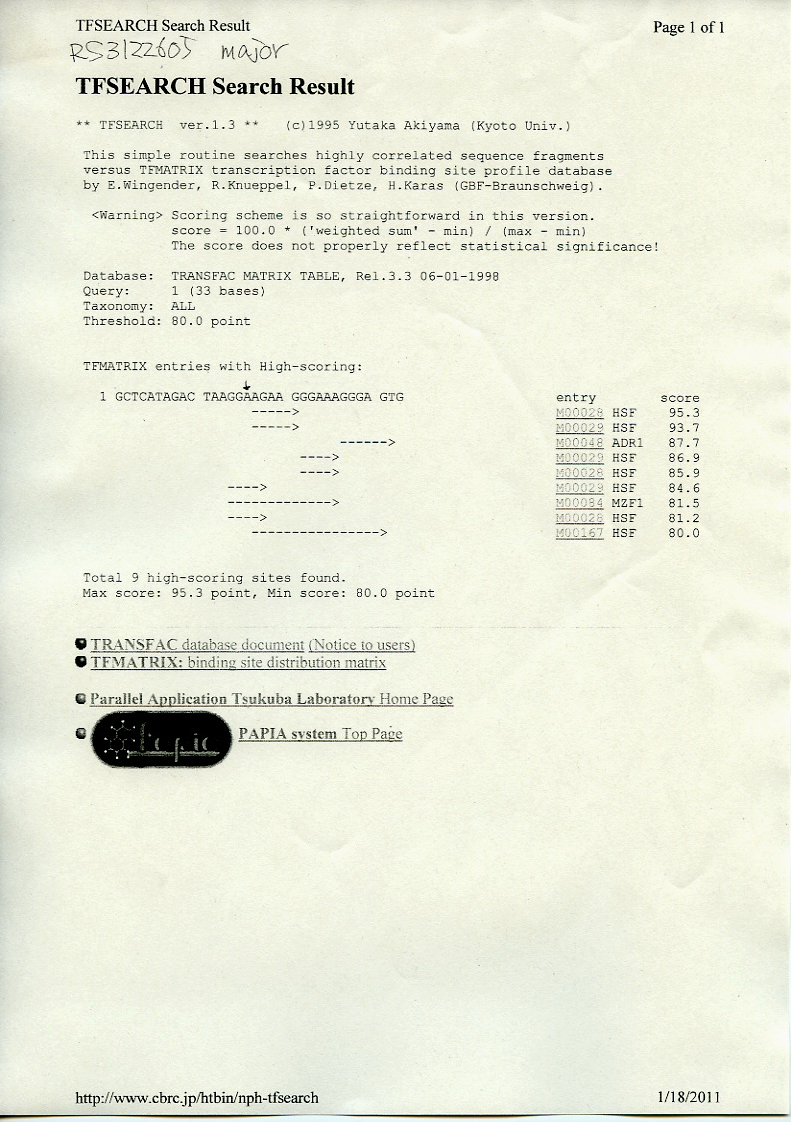

Supplement: Figure S4 — TFSEARCH search result of the major allele of rs3122605. (TIF) [file pgen.1003870.s004.tif]
