# Supplementary material for: Preferential Binding to Elk-1 by SLE-Associated IL10 Risk Allele Upregulates IL10 Expression
Source: PLoS Genet. 2013 Oct 10;9(10):e1003870. doi: 10.1371/journal.pgen.1003870 (PMC3794920; doi:10.1371/journal.pgen.1003870)
Supplement: Table S1 — Association of IL10 SNPs with SLE in European Americans. Position of each SNP is based on GRch37/hg19. Only SNPs with P<0.05 were tested in conditional testing. Four SLE-associated IL10 SNPs are highlighted in bold. Abbreviation: G, genotyped; I, imputed; ND, not distinguished; OR, odds ratio; -, missing data. (DOC) [file pgen.1003870.s005.doc]

Table S1. Association of *IL10* SNPs with SLE in European Americans

|  |  |  |  |  | Allele frequency | |  |  | *P* after conditioning on | | | |
| --- | --- | --- | --- | --- | --- | --- | --- | --- | --- | --- | --- | --- |
| Type | Annotation | SNP | Position | Tested allele | Case | Control | *P* | OR | rs3122605 | rs3024493 | rs3024495 | rs3024505 |
| I | Intergenic | rs7512090 | 206933337 | T | 5.0% | 4.6% | 0.55 | 1.05[0.89-1.24] | - | - | - | - |
| I | Intergenic | rs7519318 | 206933387 | C | 5.0% | 4.6% | 0.55 | 1.05[0.89-1.24] | - | - | - | - |
| I | Intergenic | rs7548373 | 206933490 | T | 5.0% | 4.6% | 0.55 | 1.05[0.89-1.24] | - | - | - | - |
| I | Intergenic | rs4844553 | 206934363 | T | 5.0% | 4.6% | 0.55 | 1.05[0.89-1.24] | - | - | - | - |
| I | Intergenic | rs6673928 | 206937245 | T | 22.6% | 24.3% | 0.034 | 0.92[0.85-0.99] | 0.51 | 0.37 | 0.41 | 0.34 |
| I | Intergenic | rs11119474 | 206937526 | A | 6.8% | 6.6% | 0.96 | 1.00[0.87-1.15] | - | - | - | - |
| I | Intergenic | rs61815632 | 206938439 | G | 22.6% | 24.3% | 0.034 | 0.92[0.85-0.99] | 0.52 | 0.37 | 0.41 | 0.34 |
| G | Intergenic | **rs3024505** | 206939904 | A | 18.2% | 14.8% | **2.7E-08** | 1.30[1.19-1.43] | 0.68 | ND | ND | ND |
| I | Intergenic | rs3024502 | 206940310 | T | 47.5% | 45.8% | 0.019 | 1.09[1.01-1.16] | 0.94 | 0.81 | 0.79 | 0.80 |
| I | Intergenic | rs3024500 | 206940831 | G | 47.7% | 45.9% | 0.015 | 1.09[1.02-1.17] | 0.96 | 0.83 | 0.88 | 0.89 |
| G | *IL10* | rs3024498 | 206941529 | C | 22.6% | 24.3% | 0.033 | 0.92[0.84-0.99] | 0.50 | 0.36 | 0.40 | 0.33 |
| I | *IL10* | rs3024496 | 206941864 | G | 47.6% | 45.8% | 0.017 | 1.09[1.02-1.16] | 0.97 | 0.85 | 0.83 | 0.84 |
| I | *IL10* | **rs3024495** | 206942413 | T | 17.9% | 14.8% | **1.0E-07** | 1.29[1.17-1.41] | 0.78 | ND | ND | ND |
| G | *IL10* | rs3024509 | 206943297 | G | 6.9% | 6.7% | 0.93 | 1.01[0.88-1.15] | - | - | - | - |
| I | *IL10* | rs1878672 | 206943713 | C | 47.8% | 45.9% | 0.012 | 1.09[1.02-1.17] | 0.90 | 0.87 | 0.92 | 0.94 |
| I | *IL10* | **rs3024493** | 206943968 | A | 18.0% | 14.8% | **5.0E-08** | 1.29[1.18-1.42] | 0.78 | ND | ND | ND |
| I | *IL10* | rs3024492 | 206944112 | A | 22.1% | 23.8% | 0.037 | 0.92[0.85-0.99] | 0.50 | 0.41 | 0.44 | 0.38 |
| G | *IL10* | rs1554286 | 206944233 | A | 17.2% | 18.2% | 0.13 | 0.93[0.85-1.02] | - | - | - | - |
| I | *IL10* | rs1518111 | 206944645 | T | 21.2% | 22.3% | 0.095 | 0.93[0.86-1.01] | - | - | - | - |
| I | *IL10* | rs1518110 | 206944861 | A | 21.3% | 22.5% | 0.086 | 0.93[0.86-1.01] | - | - | - | - |
| G | *IL10* | rs3021094 | 206944952 | G | 8.6% | 9.5% | 0.026 | 0.87[0.77-0.98] | 0.11 | 0.12 | 0.13 | 0.13 |
| I | *IL10* | rs3024491 | 206945046 | A | 47.6% | 45.8% | 0.017 | 1.09[1.02-1.16] | 0.97 | 0.87 | 0.84 | 0.85 |
| G | *IL10* | rs3024490 | 206945311 | A | 23.4% | 24.8% | 0.054 | 0.92[0.85-1.00] | - | - | - | - |
| G | *IL10* | rs2222202 | 206945381 | A | 47.6% | 45.8% | 0.015 | 1.09[1.02-1.17] | 0.95 | 0.93 | 0.92 | 0.91 |
| I | Intergenic | rs1800872 | 206946407 | T | 23.2% | 24.7% | 0.041 | 0.92[0.85-1.00] | 0.41 | 0.44 | 0.44 | 0.45 |
| I | Intergenic | rs1800871 | 206946634 | A | 23.2% | 24.7% | 0.039 | 0.92[0.85-1.00] | 0.40 | 0.43 | 0.43 | 0.44 |
| G | Intergenic | rs1800896 | 206946897 | C | 47.9% | 46.0% | 0.012 | 1.09[1.02-1.17] | 0.96 | 0.92 | 0.97 | 1.00 |
| I | Intergenic | rs1800893 | 206947167 | T | 47.8% | 45.9% | 0.011 | 1.09[1.02-1.17] | 0.92 | 0.92 | 0.99 | 0.99 |
| I | Intergenic | rs1800891 | 206948566 | C | 6.8% | 6.6% | 0.90 | 1.01[0.88-1.16] | - | - | - | - |
| I | Intergenic | **rs3122605** | 206955041 | G | 15.7% | 12.4% | **1.3E-08** | 1.34[1.21-1.48] | ND | 0.16 | 0.06 | 0.18 |
| I | *IL19* | rs12042283 | 206974779 | C | 38.9% | 39.6% | 0.19 | 0.95[0.89-1.02] | - | - | - | - |
| G | *IL19* | rs12040948 | 206975221 | T | 38.8% | 39.4% | 0.23 | 0.96[0.89-1.03] | - | - | - | - |
| I | *IL19* | rs61814960 | 206978249 | G | 19.2% | 20.5% | 0.054 | 0.92[0.84-1.00] | - | - | - | - |
| I | *IL19* | rs4347211 | 206978340 | G | 26.3% | 27.8% | 0.044 | 0.92[0.85-1.00] | 0.73 | 0.53 | 0.57 | 0.44 |
| I | *IL19* | rs12407461 | 206981584 | A | 25.8% | 27.1% | 0.058 | 0.93[0.86-1.00] | - | - | - | - |
| I | *IL19* | rs12407485 | 206981719 | A | 25.8% | 27.1% | 0.054 | 0.93[0.86-1.00] | - | - | - | - |
| I | *IL19* | rs17016339 | 206982066 | C | 26.3% | 27.6% | 0.063 | 0.93[0.86-1.00] | - | - | - | - |
| I | *IL19* | rs59283464 | 206982261 | G | 26.3% | 27.7% | 0.053 | 0.93[0.86-1.00] | - | - | - | - |
| I | *IL19* | rs12042745 | 206982746 | T | 26.3% | 27.7% | 0.052 | 0.93[0.86-1.00] | - | - | - | - |
| I | *IL19* | rs12409577 | 206982930 | T | 26.3% | 27.7% | 0.052 | 0.93[0.86-1.00] | - | - | - | - |
| I | *IL19* | rs10863860 | 206983365 | C | 26.3% | 27.7% | 0.053 | 0.93[0.86-1.00] | - | - | - | - |
| I | *IL19* | rs10863861 | 206983393 | T | 26.3% | 27.7% | 0.053 | 0.93[0.86-1.00] | - | - | - | - |
| I | *IL19* | rs1878673 | 206983993 | G | 26.3% | 27.7% | 0.054 | 0.93[0.86-1.00] | - | - | - | - |
| I | *IL19* | rs10863863 | 206984590 | G | 26.3% | 27.7% | 0.056 | 0.93[0.86-1.00] | - | - | - | - |
| I | *IL19* | rs11119619 | 206984811 | C | 26.3% | 27.7% | 0.056 | 0.93[0.86-1.00] | - | - | - | - |
| I | *IL19* | rs11119621 | 206985563 | G | 26.3% | 27.7% | 0.056 | 0.93[0.86-1.00] | - | - | - | - |
| I | *IL19* | rs11119622 | 206986292 | A | 26.3% | 27.7% | 0.056 | 0.93[0.86-1.00] | - | - | - | - |
| I | *IL19* | rs11119623 | 206986306 | A | 26.3% | 27.7% | 0.056 | 0.93[0.86-1.00] | - | - | - | - |
| G | *IL19* | rs7540516 | 206986545 | C | 26.3% | 27.6% | 0.054 | 0.93[0.86-1.00] | - | - | - | - |
| I | *IL19* | rs7536410 | 206986878 | T | 26.3% | 27.7% | 0.057 | 0.93[0.86-1.00] | - | - | - | - |
| I | *IL19* | rs7529836 | 206987747 | C | 26.4% | 27.7% | 0.057 | 0.93[0.86-1.00] | - | - | - | - |
| G | *IL19* | rs12044804 | 206988533 | G | 48.2% | 49.9% | 0.017 | 0.92[0.86-0.99] | 0.66 | 0.57 | 0.45 | 0.45 |
| I | *IL19* | rs12046559 | 206989067 | C | 10.5% | 10.2% | 0.69 | 1.02[0.91-1.15] | - | - | - | - |
| I | *IL19* | rs12409415 | 206989608 | T | 5.6% | 6.0% | 0.27 | 0.92[0.79-1.07] | - | - | - | - |
| I | *IL19* | rs4440852 | 206991253 | C | 20.5% | 20.8% | 0.41 | 0.96[0.88-1.05] | - | - | - | - |
| I | *IL19* | rs2883034 | 206991401 | G | 22.2% | 22.4% | 0.41 | 0.97[0.89-1.05] | - | - | - | - |
| I | *IL19* | rs72756975 | 206991584 | G | 11.9% | 11.8% | 0.89 | 0.99[0.89-1.11] | - | - | - | - |
| I | *IL19* | rs74376794 | 206992239 | A | 12.0% | 11.9% | 0.87 | 0.99[0.89-1.10] | - | - | - | - |
| I | *IL19* | rs74211061 | 206992308 | A | 12.0% | 11.9% | 0.85 | 0.99[0.89-1.10] | - | - | - | - |
| I | *IL19* | rs11119629 | 206992801 | T | 8.5% | 9.0% | 0.25 | 0.93[0.82-1.05] | - | - | - | - |
| G | *IL19* | rs2138992 | 206994404 | A | 21.4% | 21.7% | 0.33 | 0.96[0.88-1.04] | - | - | - | - |
| I | *IL19* | rs6685379 | 206995478 | G | 22.2% | 22.5% | 0.38 | 0.96[0.89-1.05] | - | - | - | - |
| I | *IL19* | rs12096695 | 206996355 | G | 8.5% | 9.0% | 0.18 | 0.92[0.81-1.04] | - | - | - | - |
| I | *IL19* | rs2056226 | 206997892 | G | 21.2% | 21.6% | 0.27 | 0.95[0.88-1.04] | - | - | - | - |
| I | *IL19* | rs2056225 | 206998706 | A | 8.5% | 9.0% | 0.17 | 0.92[0.81-1.04] | - | - | - | - |
| I | *IL19* | rs908703 | 206999963 | G | 22.3% | 22.6% | 0.39 | 0.96[0.89-1.05] | - | - | - | - |
| I | *IL19* | rs908704 | 207000060 | G | 21.3% | 21.7% | 0.27 | 0.95[0.88-1.04] | - | - | - | - |
| I | *IL19* | rs4240849 | 207000952 | A | 20.9% | 21.4% | 0.27 | 0.95[0.88-1.04] | - | - | - | - |
| I | *IL19* | rs2883035 | 207001000 | C | 21.9% | 22.3% | 0.38 | 0.96[0.89-1.05] | - | - | - | - |
| I | *IL19* | rs6540645 | 207001190 | G | 20.9% | 21.4% | 0.26 | 0.95[0.88-1.04] | - | - | - | - |
| I | *IL19* | rs1028181 | 207001709 | T | 20.9% | 21.4% | 0.27 | 0.95[0.88-1.04] | - | - | - | - |
| I | *IL19* | rs1028182 | 207001879 | T | 22.0% | 22.3% | 0.39 | 0.96[0.89-1.05] | - | - | - | - |
| G | *IL19* | rs4845143 | 207003319 | G | 22.4% | 22.5% | 0.64 | 0.98[0.90-1.07] | - | - | - | - |
| I | *IL19* | rs12022129 | 207003374 | G | 22.3% | 22.6% | 0.49 | 0.97[0.89-1.06] | - | - | - | - |
| I | *IL19* | rs6660520 | 207003553 | A | 21.1% | 21.5% | 0.31 | 0.96[0.88-1.04] | - | - | - | - |
| I | *IL19* | rs6663563 | 207003577 | T | 8.4% | 8.9% | 0.23 | 0.93[0.82-1.05] | - | - | - | - |
| I | *IL19* | rs6660537 | 207003583 | A | 8.4% | 8.9% | 0.23 | 0.93[0.82-1.05] | - | - | - | - |
| I | *IL19* | rs74148867 | 207003732 | G | 1.1% | 0.9% | 0.43 | 1.15[0.81-1.62] | - | - | - | - |
| I | *IL19* | rs7513988 | 207003956 | C | 22.7% | 22.9% | 0.52 | 0.97[0.90-1.06] | - | - | - | - |
| I | *IL19* | rs11119670 | 207005321 | C | 23.9% | 23.8% | 0.97 | 1.00[0.92-1.08] | - | - | - | - |
| I | *IL19* | rs2243156 | 207006214 | C | 8.4% | 8.8% | 0.27 | 0.93[0.83-1.05] | - | - | - | - |
| I | *IL19* | 1-207006277 | 207006277 | G | 1.1% | 0.9% | 0.43 | 1.15[0.81-1.62] | - | - | - | - |
| I | *IL19* | rs2243158 | 207007641 | C | 8.4% | 8.9% | 0.25 | 0.93[0.82-1.05] | - | - | - | - |
| I | *IL19* | rs2243168 | 207009388 | T | 8.5% | 8.9% | 0.22 | 0.93[0.82-1.05] | - | - | - | - |
| I | *IL19* | rs2243170 | 207009910 | T | 8.4% | 8.9% | 0.27 | 0.93[0.83-1.05] | - | - | - | - |
| I | *IL19* | rs2073186 | 207010626 | T | 24.3% | 24.2% | 0.94 | 1.00[0.92-1.08] | - | - | - | - |
| I | *IL19* | rs2073185 | 207010728 | A | 14.5% | 14.1% | 0.62 | 1.03[0.93-1.13] | - | - | - | - |
| I | *IL19* | rs2243171 | 207010834 | A | 8.4% | 8.9% | 0.26 | 0.93[0.83-1.05] | - | - | - | - |
| I | *IL19* | rs2243174 | 207011485 | G | 24.3% | 24.1% | 0.98 | 1.00[0.92-1.08] | - | - | - | - |
| G | *IL19* | rs2243176 | 207012444 | T | 15.3% | 14.9% | 0.52 | 1.03[0.94-1.14] | - | - | - | - |
| I | *IL19* | rs2243188 | 207014472 | A | 24.3% | 24.3% | 0.84 | 0.99[0.91-1.08] | - | - | - | - |
| G | *IL19* | rs960326 | 207014776 | C | 8.4% | 8.7% | 0.32 | 0.94[0.83-1.06] | - | - | - | - |
| I | *IL19* | rs2243191 | 207015957 | T | 22.8% | 22.9% | 0.76 | 0.99[0.91-1.07] | - | - | - | - |
| I | *IL19* | rs1798 | 207016125 | G | 15.0% | 14.9% | 0.80 | 1.01[0.92-1.12] | - | - | - | - |
| G | *IL19* | rs2243193 | 207016225 | A | 24.4% | 24.4% | 0.88 | 0.99[0.92-1.08] | - | - | - | - |
| I | Intergenic | rs4845144 | 207016648 | G | 24.3% | 24.4% | 0.81 | 0.99[0.91-1.07] | - | - | - | - |
| I | Intergenic | rs7532642 | 207018394 | T | 22.9% | 23.1% | 0.70 | 0.98[0.91-1.07] | - | - | - | - |
| I | Intergenic | rs17017001 | 207019530 | A | 14.2% | 14.2% | 0.86 | 0.99[0.90-1.10] | - | - | - | - |
| I | Intergenic | rs6671809 | 207021798 | G | 24.2% | 24.3% | 0.67 | 0.98[0.91-1.07] | - | - | - | - |
| I | Intergenic | rs4313398 | 207022139 | A | 22.9% | 23.1% | 0.69 | 0.98[0.91-1.07] | - | - | - | - |
| I | Intergenic | rs6540674 | 207023321 | A | 24.2% | 24.3% | 0.67 | 0.98[0.91-1.07] | - | - | - | - |
| I | Intergenic | rs2352797 | 207024440 | A | 24.1% | 24.3% | 0.67 | 0.98[0.91-1.07] | - | - | - | - |
| I | Intergenic | rs11119715 | 207026751 | A | 24.1% | 24.3% | 0.66 | 0.98[0.91-1.07] | - | - | - | - |
| I | Intergenic | rs1713233 | 207026835 | T | 14.2% | 14.2% | 0.86 | 0.99[0.90-1.10] | - | - | - | - |
| I | Intergenic | rs574773 | 207032751 | T | 7.2% | 7.5% | 0.33 | 0.94[0.82-1.07] | - | - | - | - |
| I | Intergenic | rs1770372 | 207035647 | C | 7.2% | 7.5% | 0.35 | 0.94[0.82-1.07] | - | - | - | - |
| I | Intergenic | rs1713239 | 207037475 | G | 13.7% | 13.7% | 0.96 | 1.00[0.90-1.11] | - | - | - | - |
| I | *IL20* | rs2981573 | 207040577 | G | 22.7% | 22.9% | 0.63 | 0.98[0.90-1.07] | - | - | - | - |
| G | *IL20* | rs2232360 | 207040659 | G | 22.9% | 23.1% | 0.63 | 0.98[0.90-1.06] | - | - | - | - |
| I | *IL24* | rs1150253 | 207071595 | A | 48.1% | 45.9% | 7.5E-03 | 1.10[1.03-1.18] | 0.10 | 0.10 | 0.09 | 0.16 |
| I | *IL24* | rs291109 | 207072151 | C | 50.4% | 47.8% | 2.0E-03 | 1.11[1.04-1.19] | 0.06 | 0.06 | 0.06 | 0.13 |
| I | *IL24* | rs291108 | 207072421 | A | 1.5% | 1.3% | 0.23 | 1.20[0.89-1.62] | - | - | - | - |
| I | *IL24* | rs1150254 | 207072515 | C | 48.1% | 45.9% | 6.7E-03 | 1.10[1.03-1.18] | 0.10 | 0.09 | 0.09 | 0.15 |
| I | *IL24* | rs1150255 | 207072959 | T | 48.1% | 45.9% | 6.7E-03 | 1.10[1.03-1.18] | 0.10 | 0.09 | 0.09 | 0.15 |
| I | *IL24* | rs1150256 | 207073133 | A | 48.1% | 45.9% | 6.7E-03 | 1.10[1.03-1.18] | 0.10 | 0.09 | 0.09 | 0.15 |
| I | *IL24* | rs1150257 | 207073491 | C | 50.4% | 47.8% | 2.2E-03 | 1.11[1.04-1.19] | 0.07 | 0.06 | 0.06 | 0.13 |
| G | *IL24* | rs1150258 | 207074905 | C | 48.1% | 45.9% | 5.9E-03 | 1.10[1.03-1.18] | 0.09 | 0.08 | 0.08 | 0.15 |
| G | *IL24* | rs291107 | 207075171 | C | 50.5% | 47.8% | 2.1E-03 | 1.11[1.04-1.19] | 0.07 | 0.06 | 0.06 | 0.13 |
| I | *FAIM3* | rs188334 | 207079615 | G | 48.1% | 45.9% | 6.2E-03 | 1.10[1.03-1.18] | 0.09 | 0.09 | 0.09 | 0.15 |
| I | *FAIM3* | rs189752 | 207079782 | G | 48.1% | 45.9% | 6.2E-03 | 1.10[1.03-1.18] | 0.09 | 0.09 | 0.09 | 0.15 |
| I | *FAIM3* | rs291106 | 207079965 | G | 48.1% | 45.9% | 6.2E-03 | 1.10[1.03-1.18] | 0.09 | 0.09 | 0.09 | 0.15 |
| I | *FAIM3* | rs291105 | 207080041 | G | 48.1% | 45.9% | 6.2E-03 | 1.10[1.03-1.18] | 0.09 | 0.09 | 0.09 | 0.15 |
| I | *FAIM3* | rs12123998 | 207080514 | C | 48.0% | 45.8% | 7.9E-03 | 1.10[1.03-1.18] | 0.12 | 0.11 | 0.11 | 0.18 |
| I | *FAIM3* | rs12121499 | 207080565 | T | 48.0% | 45.8% | 7.9E-03 | 1.10[1.03-1.18] | 0.12 | 0.11 | 0.11 | 0.18 |
| I | *FAIM3* | rs12124035 | 207080628 | A | 48.0% | 45.8% | 7.9E-03 | 1.10[1.03-1.18] | 0.12 | 0.11 | 0.11 | 0.18 |
| I | *FAIM3* | rs1713231 | 207081654 | G | 48.1% | 45.9% | 5.7E-03 | 1.10[1.03-1.18] | 0.09 | 0.09 | 0.09 | 0.15 |
| I | *FAIM3* | rs291083 | 207086969 | G | 48.6% | 46.4% | 8.0E-03 | 1.10[1.03-1.18] | 0.11 | 0.07 | 0.07 | 0.13 |
| I | *FAIM3* | rs167082 | 207087435 | T | 48.2% | 45.8% | 6.2E-03 | 1.10[1.03-1.18] | 0.09 | 0.06 | 0.06 | 0.11 |
| Abbreviation: G, genotyped; I, imputed; ND, not distinguished; OR, odds ratio; -, missing data. | | | | | | | | | | | | |
| Position of each SNP is based on GRch37/hg19. | | | | | | | | | | | | |
| Only SNPs with *P*<0.05 were tested in conditional testing. Four SLE-associated *IL10* SNPs are highlighted in bold. | | | | | | | | | | | | |
