# Supplementary material for: Preferential Binding to Elk-1 by SLE-Associated IL10 Risk Allele Upregulates IL10 Expression
Source: PLoS Genet. 2013 Oct 10;9(10):e1003870. doi: 10.1371/journal.pgen.1003870 (PMC3794920; doi:10.1371/journal.pgen.1003870)
Supplement: Table S2 — Association of IL10 cluster SNPs with SLE in Non-European ancestral groups. Position of each SNP is based on GRch37/hg19. Missing data in SNP imputation is denoted as ‘–’. Four SLE-associated SNPs identified in European Americans are highlighted in bold. Abbreviation: G, genotyped; I, imputed; OR, odds ratio. (DOC) [file pgen.1003870.s006.doc]

Table S2. Association of *IL10* cluster SNPs with SLE in Non-European ancestral groups

|  |  |  |  | African American | | | | Asian | | | | Hispanic | | | |
| --- | --- | --- | --- | --- | --- | --- | --- | --- | --- | --- | --- | --- | --- | --- | --- |
|  |  |  |  | Allele frequency | |  |  | Allele frequency | |  |  | Allele frequency | |  |  |
| Type | SNP | Position | Tested allele | Case | Control | *P* | OR | Case | Control | *P* | OR | Case | Control | *P* | OR |
| I | rs117652932 | 206933410 | A | -- | -- | -- | -- | 3.4% | 3.5% | 0.933 | 0.99[0.72-1.35] | -- | -- | -- | -- |
| I | rs4844553 | 206934363 | T | -- | -- | -- | -- | -- | -- | -- | -- | 5.0% | 5.4% | 0.971 | 0.99[0.75-1.32] |
| I | rs138307884 | 206937168 | G | 4.5% | 5.3% | 0.059 | 0.8[0.64-1.01] | -- | -- | -- | -- | -- | -- | -- | -- |
| I | rs6673928 | 206937245 | T | 11.4% | 12.2% | 0.354 | 0.93[0.8-1.08] | -- | -- | -- | -- | 13.9% | 15.2% | 0.310 | 0.91[0.77-1.09] |
| I | rs11119474 | 206937526 | A | -- | -- | -- | -- | -- | -- | -- | -- | 5.0% | 5.4% | 0.971 | 0.99[0.75-1.32] |
| I | rs61815632 | 206938439 | G | 11.4% | 12.2% | 0.354 | 0.93[0.8-1.08] | -- | -- | -- | -- | 13.9% | 15.2% | 0.310 | 0.91[0.77-1.09] |
| G | **rs3024505** | 206939904 | A | 4.5% | 3.9% | 0.251 | 1.15[0.9-1.47] | 3.1% | 2.7% | 0.375 | 1.17[0.83-1.64] | 9.1% | 8.1% | 0.054 | 1.26[1-1.58] |
| I | rs3024502 | 206940310 | T | 29.7% | 28.8% | 0.297 | 1.06[0.95-1.18] | 3.3% | 3.1% | 0.815 | 1.04[0.76-1.43] | 28.1% | 29.1% | 0.762 | 1.02[0.89-1.18] |
| I | rs12047368 | 206940523 | A | -- | -- | -- | -- | 3.4% | 3.5% | 0.933 | 0.99[0.72-1.35] | -- | -- | -- | -- |
| I | rs193120806 | 206940766 | C | -- | -- | -- | -- | 2.9% | 2.4% | 0.370 | 1.18[0.83-1.67] | -- | -- | -- | -- |
| I | rs3024500 | 206940831 | G | -- | -- | -- | -- | 6.8% | 6.8% | 0.942 | 1.01[0.81-1.26] | -- | -- | -- | -- |
| G | rs3024498 | 206941529 | C | 11.4% | 12.2% | 0.405 | 0.94[0.81-1.09] | -- | -- | -- | -- | 13.9% | 15.2% | 0.296 | 0.91[0.77-1.08] |
| I | rs3024496 | 206941864 | G | -- | -- | -- | -- | 3.3% | 3.1% | 0.822 | 1.04[0.75-1.43] | -- | -- | -- | -- |
| I | **rs3024495** | 206942413 | T | -- | -- | -- | -- | 2.9% | 2.5% | 0.430 | 1.15[0.81-1.62] | 9.0% | 8.1% | 0.045 | 1.27[1.01-1.6] |
| G | rs3024509 | 206943297 | G | 1.1% | 0.8% | 0.371 | 1.26[0.76-2.07] | -- | -- | -- | -- | 5.0% | 5.5% | 0.924 | 0.99[0.75-1.31] |
| I | rs1878672 | 206943713 | C | 30.0% | 28.9% | 0.268 | 1.06[0.95-1.18] | 6.8% | 6.8% | 0.971 | 1[0.8-1.25] | 28.4% | 29.2% | 0.700 | 1.03[0.89-1.18] |
| I | **rs3024493** | 206943968 | A | -- | -- | -- | -- | 2.9% | 2.5% | 0.436 | 1.15[0.81-1.62] | 9.0% | 8.1% | 0.051 | 1.26[1-1.59] |
| G | rs1554286 | 206944233 | A | 40.7% | 40.2% | 0.815 | 1.01[0.92-1.12] | 71.7% | 69.6% | 0.111 | 1.11[0.98-1.25] | -- | -- | -- | -- |
| I | rs1518111 | 206944645 | T | 41.2% | 40.2% | 0.509 | 1.03[0.94-1.14] | 71.3% | 69.4% | 0.148 | 1.1[0.97-1.24] | 35.2% | 33.5% | 0.973 | 1[0.88-1.14] |
| I | rs1518110 | 206944861 | A | 41.8% | 40.8% | 0.453 | 1.04[0.94-1.15] | -- | -- | -- | -- | 35.3% | 33.5% | 0.921 | 1.01[0.88-1.15] |
| G | rs3021094 | 206944952 | G | 1.7% | 1.4% | 0.402 | 1.18[0.8-1.73] | 46.2% | 44.1% | 0.125 | 1.09[0.98-1.22] | 13.6% | 12.2% | 0.525 | 1.06[0.88-1.28] |
| I | rs3024491 | 206945046 | A | 29.4% | 28.6% | 0.347 | 1.05[0.95-1.17] | 3.3% | 3.1% | 0.778 | 1.05[0.76-1.44] | 28.6% | 29.9% | 0.962 | 1[0.87-1.15] |
| G | rs3024490 | 206945311 | A | 41.8% | 40.8% | 0.452 | 1.04[0.94-1.15] | 71.1% | 69.1% | 0.113 | 1.11[0.98-1.25] | 36.2% | 34.6% | 0.992 | 1[0.88-1.14] |
| G | rs2222202 | 206945381 | A | 29.4% | 28.9% | 0.429 | 1.04[0.94-1.16] | 3.4% | 3.1% | 0.595 | 1.09[0.79-1.5] | 28.0% | 29.6% | 0.811 | 0.98[0.86-1.13] |
| I | rs3024489 | 206946244 | A | 4.5% | 5.3% | 0.052 | 0.8[0.64-1] | -- | -- | -- | -- | -- | -- | -- | -- |
| I | rs1800872 | 206946407 | T | 41.6% | 40.5% | 0.438 | 1.04[0.94-1.15] | 71.2% | 69.4% | 0.149 | 1.1[0.97-1.24] | 36.1% | 34.6% | 0.977 | 1[0.88-1.14] |
| I | rs1800871 | 206946634 | A | 41.6% | 40.5% | 0.454 | 1.04[0.94-1.15] | 71.2% | 69.4% | 0.149 | 1.1[0.97-1.24] | 36.1% | 34.6% | 0.977 | 1[0.88-1.14] |
| G | rs1800896 | 206946897 | C | 34.2% | 34.2% | 0.877 | 1.01[0.91-1.12] | 6.9% | 6.9% | 0.986 | 1[0.8-1.25] | 29.3% | 30.4% | 0.937 | 1.01[0.88-1.15] |
| I | rs1800893 | 206947167 | T | -- | -- | -- | -- | 6.8% | 6.8% | 0.962 | 1.01[0.81-1.25] | 29.3% | 30.4% | 0.906 | 1.01[0.88-1.16] |
| I | rs5743624 | 206947304 | A | 4.5% | 5.4% | 0.055 | 0.8[0.64-1] | -- | -- | -- | -- | -- | -- | -- | -- |
| I | rs12024653 | 206948542 | T | -- | -- | -- | -- | 3.6% | 3.7% | 0.971 | 0.99[0.74-1.35] | -- | -- | -- | -- |
| I | rs6693899 | 206948553 | T | -- | -- | -- | -- | 6.9% | 6.8% | 0.871 | 1.02[0.82-1.27] | -- | -- | -- | -- |
| I | rs1800891 | 206948566 | C | -- | -- | -- | -- | -- | -- | -- | -- | 5.0% | 5.5% | 0.960 | 0.99[0.75-1.32] |
| I | rs1800890 | 206949365 | T | -- | -- | -- | -- | 3.3% | 3.0% | 0.677 | 1.07[0.78-1.48] | -- | -- | -- | -- |
| I | rs34133375 | 206949402 | T | -- | -- | -- | -- | 3.6% | 3.7% | 0.966 | 0.99[0.73-1.35] | -- | -- | -- | -- |
| I | rs12026589 | 206950121 | T | -- | -- | -- | -- | 3.5% | 3.7% | 0.910 | 0.98[0.72-1.33] | -- | -- | -- | -- |
| I | rs11119514 | 206950608 | C | -- | -- | -- | -- | 6.4% | 6.4% | 0.976 | 1[0.8-1.27] | -- | -- | -- | -- |
| I | rs11119515 | 206950621 | G | -- | -- | -- | -- | 6.7% | 6.8% | 0.981 | 1[0.8-1.25] | -- | -- | -- | -- |
| I | rs6676671 | 206952748 | A | -- | -- | -- | -- | 3.0% | 2.7% | 0.560 | 1.11[0.79-1.55] | -- | -- | -- | -- |
| I | rs12123181 | 206954566 | A | -- | -- | -- | -- | 2.9% | 2.4% | 0.381 | 1.17[0.82-1.66] | -- | -- | -- | -- |
| I | **rs3122605** | 206955041 | G | -- | -- | -- | -- | 2.5% | 2.0% | 0.275 | 1.24[0.85-1.8] | -- | -- | -- | -- |
| I | rs61815643 | 206956116 | T | -- | -- | -- | -- | 2.1% | 1.5% | 0.178 | 1.34[0.87-2.06] | -- | -- | -- | -- |
| I | rs11119584 | 206973239 | G | -- | -- | -- | -- | 68.5% | 68.9% | 0.960 | 1[0.88-1.13] | -- | -- | -- | -- |
| I | rs12042283 | 206974779 | C | -- | -- | -- | -- | 68.3% | 68.2% | 0.772 | 1.02[0.9-1.15] | -- | -- | -- | -- |
| G | rs12040948 | 206975221 | T | 18.8% | 19.4% | 0.657 | 0.97[0.86-1.1] | 68.3% | 68.2% | 0.767 | 1.02[0.9-1.15] | 41.1% | 39.7% | 0.166 | 1.09[0.96-1.24] |
| I | rs4845142 | 206976233 | C | -- | -- | -- | -- | 69.0% | 69.0% | 0.813 | 1.02[0.9-1.15] | -- | -- | -- | -- |
| I | rs3950619 | 206980977 | T | -- | -- | -- | -- | 9.2% | 8.1% | 0.248 | 1.13[0.92-1.38] | -- | -- | -- | -- |
| I | rs10863859 | 206981683 | T | -- | -- | -- | -- | 9.1% | 8.1% | 0.269 | 1.12[0.92-1.37] | -- | -- | -- | -- |
| I | rs17016339 | 206982066 | C | -- | -- | -- | -- | 35.6% | 36.4% | 0.512 | 0.96[0.86-1.08] | -- | -- | -- | -- |
| I | rs59283464 | 206982261 | G | -- | -- | -- | -- | 35.6% | 36.4% | 0.512 | 0.96[0.86-1.08] | -- | -- | -- | -- |
| I | rs12042745 | 206982746 | T | 36.4% | 35.5% | 0.378 | 1.05[0.95-1.16] | -- | -- | -- | -- | 25.8% | 27.8% | 0.039 | 0.86[0.75-0.99] |
| I | rs12409577 | 206982930 | T | -- | -- | -- | -- | 35.9% | 36.8% | 0.488 | 0.96[0.86-1.08] | 25.9% | 27.9% | 0.032 | 0.86[0.75-0.99] |
| I | rs10863860 | 206983365 | C | -- | -- | -- | -- | 35.9% | 36.8% | 0.488 | 0.96[0.86-1.08] | 25.9% | 27.9% | 0.033 | 0.86[0.75-0.99] |
| I | rs10863861 | 206983393 | T | 36.0% | 35.1% | 0.395 | 1.05[0.94-1.16] | 35.9% | 36.8% | 0.488 | 0.96[0.86-1.08] | 25.8% | 27.8% | 0.036 | 0.86[0.75-0.99] |
| I | rs1878673 | 206983993 | G | 36.0% | 35.1% | 0.395 | 1.05[0.94-1.16] | 35.9% | 36.8% | 0.488 | 0.96[0.86-1.08] | 25.8% | 27.8% | 0.036 | 0.86[0.75-0.99] |
| I | rs10863863 | 206984590 | G | -- | -- | -- | -- | 35.9% | 36.8% | 0.488 | 0.96[0.86-1.08] | 25.9% | 27.9% | 0.033 | 0.86[0.75-0.99] |
| I | rs11119619 | 206984811 | C | -- | -- | -- | -- | 35.9% | 36.8% | 0.488 | 0.96[0.86-1.08] | 25.9% | 27.9% | 0.033 | 0.86[0.75-0.99] |
| I | rs11119621 | 206985563 | G | -- | -- | -- | -- | 35.9% | 36.8% | 0.488 | 0.96[0.86-1.08] | 25.6% | 27.6% | 0.032 | 0.86[0.74-0.99] |
| I | rs11119622 | 206986292 | A | 35.9% | 35.0% | 0.390 | 1.05[0.94-1.16] | 35.9% | 36.8% | 0.488 | 0.96[0.86-1.08] | 25.8% | 27.8% | 0.036 | 0.86[0.75-0.99] |
| I | rs11119623 | 206986306 | A | 35.9% | 35.0% | 0.390 | 1.05[0.94-1.16] | 35.9% | 36.8% | 0.488 | 0.96[0.86-1.08] | 25.8% | 27.8% | 0.036 | 0.86[0.75-0.99] |
| G | rs7540516 | 206986545 | C | 35.9% | 34.9% | 0.332 | 1.05[0.95-1.16] | 35.7% | 36.8% | 0.411 | 0.95[0.85-1.07] | 25.8% | 27.7% | 0.045 | 0.87[0.75-1] |
| I | rs7536410 | 206986878 | T | 35.7% | 34.9% | 0.383 | 1.05[0.95-1.16] | 35.9% | 36.8% | 0.488 | 0.96[0.86-1.08] | 25.8% | 27.8% | 0.036 | 0.86[0.75-0.99] |
| I | rs7521798 | 206986926 | T | 50.7% | 50.0% | 0.556 | 1.03[0.93-1.14] | 90.5% | 91.7% | 0.210 | 0.88[0.72-1.08] | -- | -- | -- | -- |
| I | rs7529836 | 206987747 | C | -- | -- | -- | -- | 35.9% | 36.8% | 0.469 | 0.96[0.86-1.08] | 25.7% | 27.7% | 0.037 | 0.86[0.75-0.99] |
| G | rs12044804 | 206988533 | G | 51.8% | 50.9% | 0.442 | 1.04[0.94-1.15] | 88.9% | 89.5% | 0.578 | 0.95[0.8-1.14] | 55.6% | 54.9% | 0.878 | 0.99[0.87-1.13] |
| I | rs2883034 | 206991401 | G | -- | -- | -- | -- | -- | -- | -- | -- | 36.5% | 36.1% | 0.546 | 0.96[0.84-1.1] |
| I | rs74211061 | 206992308 | A | 2.0% | 2.2% | 0.544 | 0.9[0.64-1.26] | -- | -- | -- | -- | 18.7% | 18.6% | 0.592 | 0.96[0.81-1.13] |
| G | rs2138992 | 206994404 | A | 35.3% | 34.8% | 0.482 | 1.04[0.94-1.15] | 63.1% | 63.0% | 0.989 | 1[0.89-1.12] | 36.0% | 35.6% | 0.513 | 0.96[0.84-1.09] |
| I | rs6685379 | 206995478 | G | -- | -- | -- | -- | 62.9% | 62.9% | 0.939 | 1[0.89-1.12] | 36.1% | 36.1% | 0.401 | 0.94[0.83-1.08] |
| I | rs79798148 | 206997561 | A | -- | -- | -- | -- | -- | -- | -- | -- | -- | -- | -- | -- |
| I | rs2056226 | 206997892 | G | -- | -- | -- | -- | 62.6% | 62.7% | 0.889 | 0.99[0.89-1.11] | 34.7% | 34.5% | 0.441 | 0.95[0.83-1.09] |
| I | rs908703 | 206999963 | G | -- | -- | -- | -- | 63.0% | 62.9% | 0.988 | 1[0.89-1.12] | 36.2% | 36.1% | 0.408 | 0.95[0.83-1.08] |
| I | rs908704 | 207000060 | G | 34.1% | 33.9% | 0.662 | 1.02[0.92-1.14] | 63.0% | 62.9% | 0.988 | 1[0.89-1.12] | 35.4% | 35.2% | 0.452 | 0.95[0.83-1.09] |
| I | rs4240849 | 207000952 | A | -- | -- | -- | -- | 61.5% | 61.6% | 0.848 | 0.99[0.88-1.11] | -- | -- | -- | -- |
| I | rs6540645 | 207001190 | G | -- | -- | -- | -- | 62.7% | 62.7% | 0.945 | 1[0.89-1.12] | 34.5% | 34.4% | 0.416 | 0.95[0.83-1.08] |
| I | rs1028181 | 207001709 | T | -- | -- | -- | -- | 62.7% | 62.7% | 0.945 | 1[0.89-1.12] | 34.6% | 34.2% | 0.469 | 0.95[0.83-1.09] |
| I | rs1028182 | 207001879 | T | -- | -- | -- | -- | 63.0% | 62.9% | 0.985 | 1[0.89-1.12] | 36.5% | 36.6% | 0.315 | 0.93[0.82-1.07] |
| G | rs4845143 | 207003319 | G | 34.3% | 33.7% | 0.409 | 1.04[0.94-1.16] | 62.4% | 62.3% | 0.979 | 1[0.89-1.12] | 35.7% | 35.7% | 0.348 | 0.94[0.82-1.07] |
| I | rs12022129 | 207003374 | G | -- | -- | -- | -- | 62.5% | 62.3% | 0.968 | 1[0.9-1.12] | 36.5% | 36.4% | 0.348 | 0.94[0.82-1.07] |
| I | rs6660520 | 207003553 | A | -- | -- | -- | -- | 62.8% | 62.6% | 0.901 | 1.01[0.9-1.13] | 35.6% | 35.3% | 0.433 | 0.95[0.83-1.08] |
| I | rs6663563 | 207003577 | T | -- | -- | -- | -- | -- | -- | -- | -- | 14.3% | 13.8% | 0.930 | 0.99[0.83-1.19] |
| I | rs6660537 | 207003583 | A | -- | -- | -- | -- | -- | -- | -- | -- | 14.3% | 13.8% | 0.930 | 0.99[0.83-1.19] |
| I | rs7513988 | 207003956 | C | -- | -- | -- | -- | 62.8% | 62.6% | 0.901 | 1.01[0.9-1.13] | 36.5% | 36.5% | 0.338 | 0.94[0.82-1.07] |
| I | rs11119670 | 207005321 | C | -- | -- | -- | -- | 69.5% | 68.5% | 0.548 | 1.04[0.92-1.17] | -- | -- | -- | -- |
| I | rs2243156 | 207006214 | C | -- | -- | -- | -- | 24.5% | 24.8% | 0.778 | 0.98[0.86-1.12] | 13.5% | 13.0% | 0.862 | 0.98[0.82-1.18] |
| I | rs2243158 | 207007641 | C | -- | -- | -- | -- | 24.6% | 25.1% | 0.674 | 0.97[0.85-1.11] | 13.5% | 13.0% | 0.862 | 0.98[0.82-1.18] |
| I | rs2243168 | 207009388 | T | 30.4% | 30.1% | 0.676 | 1.02[0.92-1.14] | 24.6% | 24.8% | 0.803 | 0.98[0.86-1.12] | 14.5% | 14.3% | 0.705 | 0.97[0.81-1.15] |
| I | rs2243170 | 207009910 | T | 30.4% | 30.1% | 0.676 | 1.02[0.92-1.14] | 24.6% | 24.8% | 0.803 | 0.98[0.86-1.12] | 14.5% | 14.3% | 0.705 | 0.97[0.81-1.15] |
| I | rs2073186 | 207010626 | T | 40.8% | 40.3% | 0.350 | 1.05[0.95-1.16] | 69.5% | 68.5% | 0.527 | 1.04[0.92-1.17] | 36.2% | 37.6% | 0.065 | 0.88[0.77-1.01] |
| I | rs2073185 | 207010728 | A | 2.7% | 2.9% | 0.563 | 0.92[0.68-1.23] | 44.0% | 42.8% | 0.462 | 1.04[0.93-1.17] | 19.7% | 21.1% | 0.106 | 0.88[0.75-1.03] |
| I | rs2243171 | 207010834 | A | 30.4% | 30.1% | 0.676 | 1.02[0.92-1.14] | 24.6% | 24.8% | 0.803 | 0.98[0.86-1.12] | 14.5% | 14.3% | 0.705 | 0.97[0.81-1.15] |
| I | rs2243174 | 207011485 | G | 41.0% | 40.3% | 0.299 | 1.06[0.95-1.17] | 69.5% | 68.6% | 0.542 | 1.04[0.92-1.17] | 36.3% | 37.7% | 0.077 | 0.89[0.78-1.01] |
| G | rs2243176 | 207012444 | T | 11.2% | 10.9% | 0.464 | 1.06[0.91-1.23] | 44.6% | 43.5% | 0.486 | 1.04[0.93-1.16] | 21.1% | 22.4% | 0.143 | 0.89[0.77-1.04] |
| I | rs2243188 | 207014472 | A | 42.1% | 41.7% | 0.443 | 1.04[0.94-1.15] | 69.5% | 68.6% | 0.529 | 1.04[0.92-1.17] | 36.1% | 37.7% | 0.069 | 0.89[0.78-1.01] |
| G | rs960326 | 207014776 | C | 31.2% | 31.0% | 0.801 | 1.01[0.91-1.13] | 25.2% | 24.9% | 0.850 | 1.01[0.89-1.15] | 14.5% | 14.4% | 0.663 | 0.96[0.81-1.15] |
| G | rs2243193 | 207016225 | A | 43.2% | 43.2% | 0.753 | 1.02[0.92-1.12] | 76.8% | 76.6% | 0.852 | 1.01[0.89-1.16] | 36.7% | 37.9% | 0.100 | 0.9[0.79-1.02] |
| I | rs4845144 | 207016648 | G | 41.6% | 41.2% | 0.520 | 1.03[0.94-1.14] | 77.1% | 76.6% | 0.709 | 1.03[0.9-1.17] | 36.5% | 37.7% | 0.105 | 0.9[0.79-1.02] |
| I | rs7532642 | 207018394 | T | -- | -- | -- | -- | 76.5% | 76.2% | 0.815 | 1.02[0.89-1.16] | 34.9% | 36.2% | 0.088 | 0.89[0.78-1.02] |
| I | rs6671809 | 207021798 | G | 41.6% | 41.2% | 0.517 | 1.03[0.94-1.14] | -- | -- | -- | -- | 37.0% | 38.2% | 0.092 | 0.89[0.78-1.02] |
| I | rs4313398 | 207022139 | A | -- | -- | -- | -- | 76.6% | 76.2% | 0.792 | 1.02[0.89-1.16] | 35.2% | 36.3% | 0.116 | 0.9[0.79-1.03] |
| I | rs6540674 | 207023321 | A | 41.6% | 41.3% | 0.549 | 1.03[0.93-1.14] | -- | -- | -- | -- | 37.0% | 38.2% | 0.092 | 0.89[0.78-1.02] |
| I | rs2352797 | 207024440 | A | 41.6% | 41.3% | 0.549 | 1.03[0.93-1.14] | -- | -- | -- | -- | 37.0% | 38.2% | 0.092 | 0.89[0.78-1.02] |
| I | rs11119715 | 207026751 | A | 41.6% | 41.3% | 0.549 | 1.03[0.93-1.14] | -- | -- | -- | -- | 37.0% | 38.2% | 0.092 | 0.89[0.78-1.02] |
| I | rs1770372 | 207035647 | C | 13.4% | 13.8% | 0.609 | 0.96[0.83-1.11] | -- | -- | -- | -- | -- | -- | -- | -- |
| I | rs2981573 | 207040577 | G | 16.7% | 17.2% | 0.525 | 0.96[0.84-1.09] | 76.2% | 76.2% | 0.971 | 1[0.87-1.14] | 33.9% | 35.1% | 0.093 | 0.89[0.78-1.02] |
| G | rs2232360 | 207040659 | G | 17.4% | 18.0% | 0.482 | 0.96[0.84-1.09] | 75.7% | 75.9% | 0.854 | 0.99[0.87-1.13] | 33.7% | 35.1% | 0.073 | 0.89[0.78-1.01] |
| I | rs574635 | 207064391 | G | 81.0% | 80.5% | 0.637 | 1.03[0.91-1.17] | -- | -- | -- | -- | -- | -- | -- | -- |
| I | rs3860300 | 207064918 | G | -- | -- | -- | -- | 18.7% | 20.1% | 0.179 | 0.9[0.78-1.05] | -- | -- | -- | -- |
| I | rs6540703 | 207065806 | A | -- | -- | -- | -- | 18.7% | 20.1% | 0.179 | 0.9[0.78-1.05] | -- | -- | -- | -- |
| I | rs4845147 | 207066383 | T | -- | -- | -- | -- | 18.7% | 20.1% | 0.202 | 0.91[0.78-1.05] | -- | -- | -- | -- |
| I | rs569011 | 207066559 | A | 79.0% | 79.3% | 0.866 | 0.99[0.88-1.12] | -- | -- | -- | -- | -- | -- | -- | -- |
| I | rs1624793 | 207067194 | G | 79.0% | 79.3% | 0.866 | 0.99[0.88-1.12] | -- | -- | -- | -- | -- | -- | -- | -- |
| I | rs1624787 | 207067199 | G | 79.0% | 79.3% | 0.866 | 0.99[0.88-1.12] | -- | -- | -- | -- | -- | -- | -- | -- |
| I | rs291112 | 207067372 | G | 79.0% | 79.3% | 0.866 | 0.99[0.88-1.12] | -- | -- | -- | -- | -- | -- | -- | -- |
| I | rs1850570 | 207068282 | T | -- | -- | -- | -- | 18.7% | 20.1% | 0.202 | 0.91[0.78-1.05] | -- | -- | -- | -- |
| I | rs3762344 | 207068672 | A | -- | -- | -- | -- | 18.7% | 20.1% | 0.202 | 0.91[0.78-1.05] | -- | -- | -- | -- |
| I | rs1150253 | 207071595 | A | -- | -- | -- | -- | 20.1% | 21.2% | 0.280 | 0.92[0.8-1.07] | 45.9% | 43.3% | 0.096 | 1.11[0.98-1.26] |
| I | rs291109 | 207072151 | C | 79.0% | 79.3% | 0.866 | 0.99[0.88-1.12] | 21.2% | 22.3% | 0.282 | 0.93[0.8-1.07] | 51.3% | 49.4% | 0.226 | 1.08[0.95-1.22] |
| I | rs1150254 | 207072515 | C | 28.1% | 28.5% | 0.790 | 0.99[0.88-1.1] | 20.7% | 21.5% | 0.411 | 0.94[0.82-1.09] | 45.9% | 43.3% | 0.096 | 1.11[0.98-1.26] |
| I | rs1150255 | 207072959 | T | 27.9% | 27.7% | 0.832 | 1.01[0.91-1.13] | 20.7% | 21.5% | 0.411 | 0.94[0.82-1.09] | 45.9% | 43.3% | 0.096 | 1.11[0.98-1.26] |
| I | rs1150256 | 207073133 | A | 27.9% | 27.7% | 0.832 | 1.01[0.91-1.13] | 20.7% | 21.5% | 0.411 | 0.94[0.82-1.09] | 45.9% | 43.3% | 0.096 | 1.11[0.98-1.26] |
| I | rs1150257 | 207073491 | C | 79.0% | 79.3% | 0.866 | 0.99[0.88-1.12] | 21.4% | 22.5% | 0.308 | 0.93[0.81-1.07] | 51.3% | 49.4% | 0.226 | 1.08[0.95-1.22] |
| G | rs1150258 | 207074905 | C | 27.9% | 27.7% | 0.818 | 1.01[0.91-1.13] | 20.8% | 21.5% | 0.407 | 0.94[0.82-1.08] | 45.9% | 43.3% | 0.102 | 1.11[0.98-1.26] |
| G | rs291107 | 207075171 | C | 79.0% | 79.3% | 0.812 | 0.99[0.87-1.11] | 22.3% | 23.1% | 0.411 | 0.94[0.82-1.08] | 51.3% | 49.4% | 0.235 | 1.08[0.95-1.22] |
| Abbreviation: G, genotyped; I, imputed; OR, odds ratio.  Position of each SNP is based on GRch37/hg19.  Missing data in SNP imputation is denoted as '--'. Four SLE-associated SNPs identified in European Americans are highlighted in bold. | | | | | | | | | | | | | | | |
|
