# Supplementary material for: Preferential Binding to Elk-1 by SLE-Associated IL10 Risk Allele Upregulates IL10 Expression
Source: PLoS Genet. 2013 Oct 10;9(10):e1003870. doi: 10.1371/journal.pgen.1003870 (PMC3794920; doi:10.1371/journal.pgen.1003870)
Supplement: Table S3 — DNA sequences of oligodeoxynucleotide probes used in EMSA. (DOC) [file pgen.1003870.s007.doc]

Table S3: DNA sequences of oligodeoxynucleotide probes used in EMSA

| **SNP** | **Strand** | **DNA sequence** |
| --- | --- | --- |
| rs3024505 | Forward | 5' GCA GAG CGT GAG GG(**G/A**)GA CTA GTG TTT ACT |
|  | Reverse | 5' AGT AAA CAC TAG TC(**C/T**)CC CTC ACG CTC TGC |
| rs3024495 | Forward | 5' CTT CGA AAG CAA GA(**C/T**)GG TGA GAG GAG AGG |
|  | Reverse | 5' CCT CTC CTC TCA CC(**G/A**)TC TTG CTT TCG AAG |
| rs3024493 | Forward | 5' ACT GAA GCT CTG GG(**C/A**)TC CTT TTA TGA AGA |
|  | Reverse | 5' TCT TCA TAA AAG GA(**G/T**)CC CAG AGC TTC AGT |
| rs3122605 | Forward | 5' CTC ATA GAC TAA GG(**A/G)**AG AAG GGA AAG GGA |
|  | Reverse | 5' TCC CTT TCC CTT CT(**T/C)**CC TTA GTC TAT GAG |
| rs61815643 | Forward | 5’ CTA GAG GCT G(**G/T**)G TGT GGT CAA |
|  | Reverse | 5’ TTG ACC ACA C(**C/A**)C AGC CTC TAG |
